# Characterization of aroma profiles and microbial communities of cigar tobacco leaves from different varieties and origins and their correlations analysis

**DOI:** 10.1038/s41598-025-07310-0

**Published:** 2025-07-15

**Authors:** Zhaoliang Geng, Huajun Gao, Zhuokuan Tang, Wenhui Zhu, Tongjing Yan, Beisen Lin, Qi Li, Xianwei Hao, Can Lyu, Bin Cai, Zelin He, Jian Liu

**Affiliations:** 1 Hainan Provincial Branch of China National Tobacco Corporation, Haikou, 571103 China; 2 Hainan Hongta Cigarette Company Limited, Haikou, 571137 China; 3 China Tobacco Zhejiang Industrial Company Limited, Hangzhou, 311500 China; 4 Institute of Tobacco Research, Chinese Academy of Agricultural Sciences, Qingdao, 266101 China; 5 Beijing Life Science Academy, Beijing, 100085 China

**Keywords:** Cigar, Aroma, Microbial community, Variety, Origin, Microbiology, Plant sciences

## Abstract

Microorganisms play an important role in cigar tobacco leaves (CTLs) production, while the impact of varieties and origins on microorganisms and thereby on aroma profiles were rarely reported. In this study, the aroma profiles of wrapper and filler CTLs from three varieties, respectively, and filler CTLs from four origins were analyzed, and the bacterial and fungal communities of these CTLs and their correlation with aroma constituents were explored. The results showed that ketones and nitrogen heterocycles were dominant in wrapper and filler CTLs of different varieties and filler CTLs from different origins, their proportions in different CTLs were 22.34–58.28% and 19.45–53.62%, respectively. There were significant differences in the varieties and contents of aroma constituents in CTLs of different varieties and origins, which showed obvious variety and origin specificity. For example, the contents of aldehydes (92.24 μg/kg), ketones (1553.00 μg/kg), and alkenes (227.14 μg/kg) in the wrapper variety of AQ2 increased significantly, the contents of ketones (2027.21 μg/kg), alkenes (219.98 μg/kg), and total aroma constituents (4262.60 μg/kg) in the filler variety of QDQX2 increased, and the contents of ketones, nitrogen heterocycles, alkenes, and total aroma constituents in the origin of FXQX1 increased, which increased by 0.29–0.84, 0.44–0.96, 0.22–7.19, and 0.34–0.65 times. Besides, the microbial community diversity and structure were significantly affected by varieties and origins. There were obvious differences in the α- and β-diversity of microbial communities in different varieties and origins of CTLs. Varieties have little effect on the bacterial communities of wrapper and filler CTLs, and only the abundances of the dominant *Corynebacterium* (45.78–55.34% of wrapper and 29.97–60.90% of filler) and *Staphylococcus* (15.16–34.60% of wrapper and 22.12–46.70% of filler) were different, but there were significant differences in the composition and abundance of the fungal community. Different from the influence of varieties, the composition and abundance of bacterial communities were significantly changed by the origins, while only the abundances of the dominant *Alternaria* (6.45–46.73%), *Aspergillus* (5.72–32.24%), *Wallemia* (11.80–24.84%), and *Cladosporium* (1.12–10.89%) in the fungal community were different. Correlation analyses showed that different bacterial and fungal communities synergistically contributed to the formation of aroma profiles of CTLs, and the microbial communities contributing to the formation of aroma profiles were obviously different among wrapper and filler varieties and filler origins.

**Supplementary Information:**

The online version contains supplementary material available at 10.1038/s41598-025-07310-0.

## Introduction

The cigar is renowned for its profound cultural heritage and exceptional taste^1^. Its flavor is characterized by a strong, mellow, and varied when compared with flue-cured tobacco, which exhibits a range of aromas including fruity, floral, nutty, chocolate, coffee, and milk^2^. A cigar is a hand-rolled tobacco product comprising filler, binder, and wrapper. Its sensory characteristics are closely related to the aroma profile of tobacco leaves. Flavor is the fundamental attribute of cigar tobacco leaves (CTLs), determining the quality and style characteristics^3^. The chemical constituents of CTLs, in terms of their composition, content, and balance, serve as the basis for establishing the flavor profile. These chemical constituent profiles are influenced by a range of factors, including variety, planting environment, and production technology.

The elaboration of a cigar encompasses a series of processes, including cultivation, air-curing, fermentation, and aging. These processes, along with the accumulation, degradation, and transformation of macromolecules and the corresponding biosynthesis of aroma precursors and volatile flavor compounds, are influenced by the growth and metabolism of microorganisms^4–6^. Microorganisms can regulate metabolic levels through the production of specific signaling molecules, which in turn affect the expression of genes associated with metabolites in plants, alternatively, they may participate in synthetic signaling pathways^7–10^. The distinction in the structure and functionality of the microbial community gives rise to variations in flavor^11^. Consequently, a thorough comprehension of the microbial community and its role is of paramount importance in regulating cigar flavor^12,13^.

Right now, the roles of microorganisms in forming aroma profiles during air-curing and fermentation are well documented^14^. Bacteria are mainly involved in sugar metabolism, lipid metabolism, and amino acid metabolism, while fungi are mainly involved in saprophytic degradation of lignin, cellulose, and pectin^15,16^. For example, the microbial communities in the middle CTLs had higher abundances of metabolic pathways related to carbohydrates and amino acids than those in the upper CTLs, potentially leading to the formation of more flavor compounds during air-curing^17^. The predominant bacteria in cigars hold significant potential for applications in the degradation of aromatic compounds, hydrocarbons, aliphatic compounds, and lignin, while fungi primarily served a saprotrophic role after fermentation^18^. The starch in wrapper, binder, and filler CTLs has a significant positive effect on fungal microbes and thus indirectly on the formation of nitrogenous flavor compounds^19^. Besides, the influence of functional microorganisms, fermentation media, and origins on the microbial communities and corresponding aroma profile of CTLs are also analyzed. For example, inoculation with *Staphylococcus nepalensis* resulted in higher consumption rates of reducing sugars and total nitrogen contents during the CTL fermentation, and it was positively correlated with carotenoid degradation products, indicating its potential role in promoting flavor formation^20^. *Bacillus siamensis* inoculation during CTL fermentation resulted in a significant enrichment of *Pseudomonas*, which played an important role in sugar metabolism and pectin degradation^21^. The microbial functions of cigar filler leaves supplemented with the *Tremella aurantialba* SCT-F3 fermentation medium were enhanced, including nucleotide biosynthesis, amino acid biosynthesis, fatty acid and lipid biosynthesis, nicotine degradation, and nicotinate degradation^22^.

The tobacco aroma quality is mainly determined by the genetic factors of the variety and the environmental factors of the origin. The variety influences the nature and variety of aroma constituents, while the origin mainly affects the content and composition of aroma constituents. The breeding and selection of superior varieties is a fundamental prerequisite to producing tobacco leaves with an exceptional aroma profile^23^. The variety has a direct impact on the quality of the tobacco leaf, influencing gene expression regulation. The differences in genotype and genetic basis result in varying contents of aroma constituents among the various tobacco leaf varieties. It is challenging to rectify the deficiencies in the aroma profile of tobacco varieties through cultivation methods alone^24^. Biological factors exert a considerable influence on the concentration of plant metabolites. However, there is a paucity of literature examining the regulation of cigar flavor both from the perspective of varieties and microorganisms.

Flue-cured tobacco is very sensitive to the ecological environment, and the ecological conditions of the origin basically determine the flavor profiles and quality of the tobacco and form the basis of the style of different brands of cigarettes. Many tobacco origins have been declared as protected geographical indications to establish and maintain the characteristics and quality of the tobacco from which they originate. Comprehensively analyzing the composition and characteristics of aroma constituents in tobacco from different origins helps recognize the characteristics of tobacco, trace the origin of tobacco, maintain geographical indications, protect the rights and interests of tobacco farmers, and regulate the quality of tobacco. Besides, the microbial communities were changed among different origins, thereby affecting the aroma profile of CTLs.

The current study was carried out to investigate the effect of the filler and wrapper from three varieties and the filler from four origins on the aroma profile of CTLs. The key aroma constituents to these CTLs and the difference in the microbial community diversity and structure were explored. The relationship between both dominant microorganisms and aroma constituents was also revealed. It laid a foundation for the efficient regulation of the aroma quality of cigars.

## Results

### Effect of variety on aroma profiles and differential constituents of wrapper CTLs

**Fig. 1:**
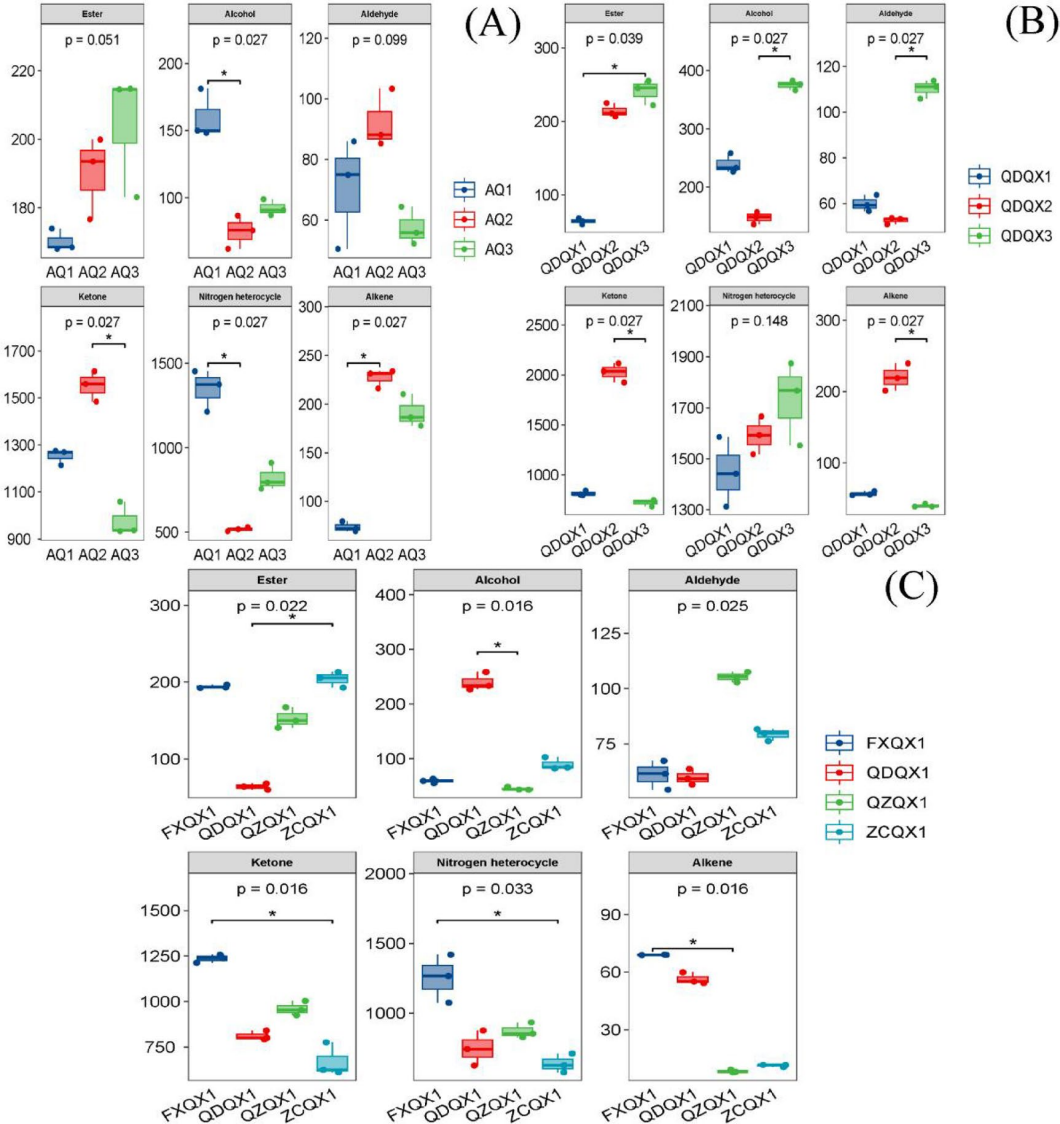
The aroma profiles of cigar tobacco leaves (n = 3). (**A**) Wrapper of different varieties. (**B**) Filler of different varieties. (**C**) Filler of different origins.

A total of 60 aroma constituents were detected in three varieties of wrapper CTLs, which were divided into 7 classes according to their chemical structures, including esters (6), alcohols (6), acids (1), aldehydes (7), ketones (19), nitrogen heterocycles (13), and alkenes (8), and their total content was between 2.40 mg/kg and 3.08 mg/kg (Table S1). There were significant differences in the varieties and contents of aroma constituents, and the changes in aroma constituents exhibited obvious variety specificity. Among them, the contents of alcohols, nitrogen heterocycles, and total aroma constituents in AQ1 increased, the aldehydes, ketones, and alkenes in AQ2 enhanced, and the esters in AQ3 increased (Fig. 1A). Besides, there were significant differences in the proportions of the aroma profile between different varieties of wrapper CTLs, including the esters (5.53–8.50%), alcohols (2.81–5.20%), nitrogen heterocycles (19.45–43.82%), and alkenes (2.40–8.52%). Volatiles were one of the most important factors in terms of aroma and taste, especially in tobacco, where many volatile compounds could be transferred from the tobacco to the smoke by volatilization without any structural changes^25^. Therefore, cigar varieties could be determined by their volatiles.

**Fig. 2:**
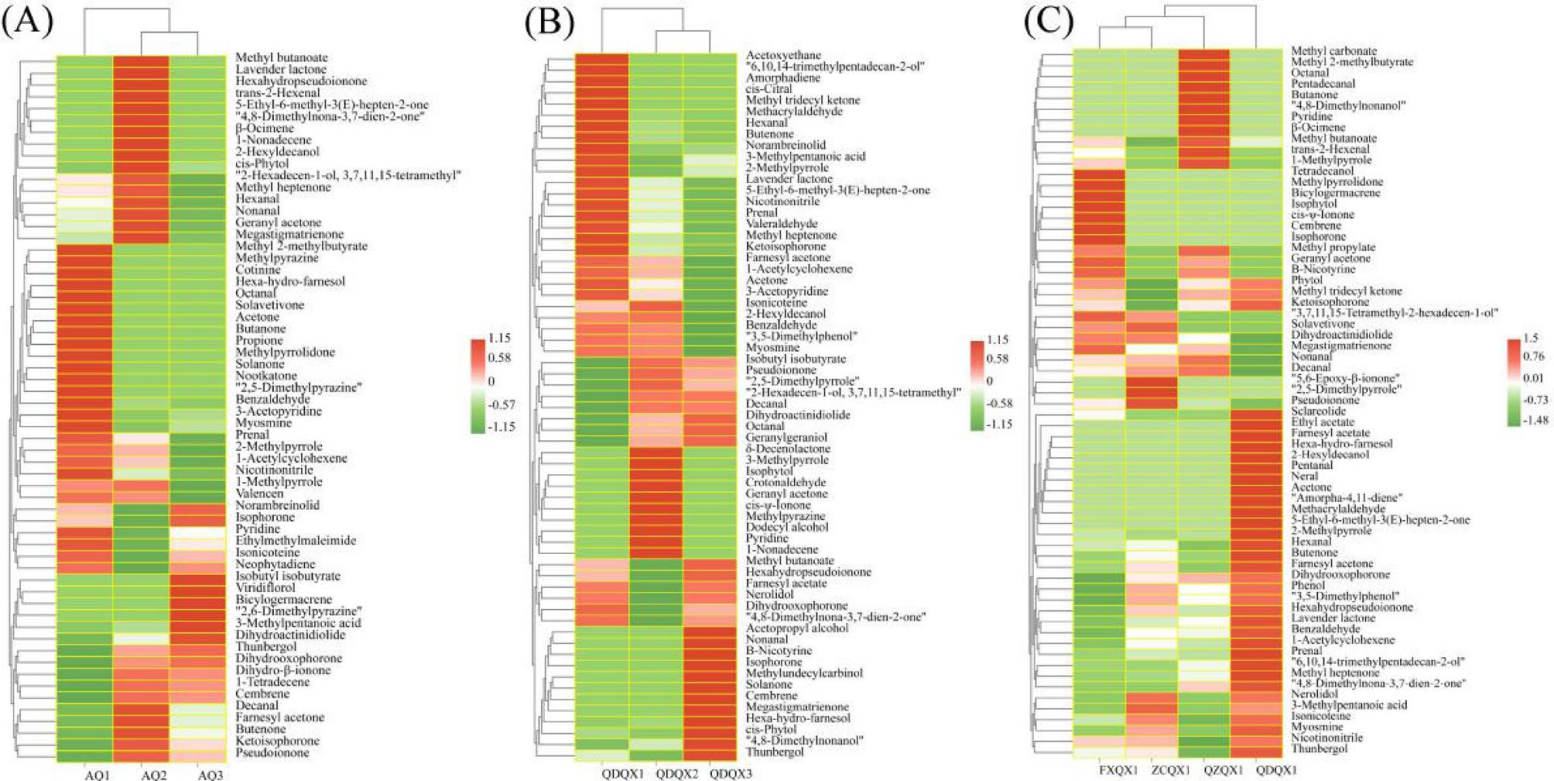
The heatmap cluster of aroma constituents of cigar tobacco leaves. (**A**) Wrapper of different varieties. (**B**) Filler of different varieties. (**C**) Filler of different origins.

Esters provide sweet and fruity flavors to tobacco^26^. The total esters content of AQ3 increased by 20.01% compared with AQ1, due to isobutyl isobutyrate was newly detected (Fig. 2A), and dihydroactinidiolide and norambreinolid increased by 18.99% and 38.33%, respectively. Methyl 2-methylbutyrate and lavender lactone were newly detected in AQ1 and AQ2. Alcohols help to enhance the floral and fruity aromas of tobacco^27^. The total alcohols content of AQ1 increased significantly, as the content of newly detected hexa-hydro-farnesol reached 107.01 μg/kg. 2-Hexyldecanol was newly detected and the contents of 3,7,11,15-tetramethyl-2-hexadecen-1-ol and thunbergol were significantly increased in AQ2. Viridiflorol was newly found and the content of 3-methylpentanoic acid increased in AQ3. The total aldehydes content of AQ2 increased significantly, due to the newly detected trans-2-hexenal, and the contents of hexanal and decanal increased to 6.45 μg/kg and 16.14 μg/kg, respectively. Octanal was newly found and the contents of prenal and benzaldehyde increased in AQ1.

Ketones could form important aromatic molecules through carotenoid degradation^28^. The total ketones content of AQ2 reached 1.56 mg/kg, which was 23.87% and 59.07% higher than AQ1 and AQ3, respectively, due to the newly detected 5-ethyl-6-methyl-3(E)-hepten-2-one, 4,8-dimethylnona-3,7-dien-2-one, and hexahydropseudoionone, the content of hexahydropseudoionone reached 111.28 μg/kg. The contents of butenone, methyl heptenone, ketoisophorone, dihydro-β-ionone, pseudoionone, geranyl acetone, and megastigmatrienone also increased significantly, and geranyl acetone and megastigmatrienone reached 978.89 μg/kg and 181.27 μg/kg, respectively, the former increased by 21.90% and 40.22% compared with AQ1 and AQ3, and the latter increased by 1.63 and 5.10 times, respectively. Megastigmatrienone mainly imparted woody and floral flavors to tobacco^29^. Acetone, butanone, propione, solanone, nootkatone, and solavetivone were newly detected in AQ1.

The total nitrogen heterocycles content of AQ1 increased significantly, reaching 1.35 mg/kg, which was 1.61 and 0.64 times higher than AQ2 and AQ3, respectively, mainly due to the newly detected methylpyrazine, 2,5-dimethylpyrazine, methylpyrrolidone, cotinine, and pyridine, and the contents of 2-methylpyrrole, nicotinonitrile, 3-acetopyridine, ethylmethylmaleimide, and myosmine increased significantly. The contents of 3-acetopyridine and myosmine reached 588.41 μg/kg and 335.63 μg/kg, respectively, the former increased by 53.81% and 43.22% compared with AQ2 and AQ3, while the latter increased by 2.70 and 1.40 times, respectively. Compounds derived from N-heterocyclic carbenes such as pyridine play a key role in shaping the aroma of cigars^30^. The total alkenes content of AQ2 reached 227.14 μg/kg and increased by 208.22% and 18.57% compared with AQ1 and AQ3, respectively, as the newly detected β-ocimene and 1-nonadecene. The contents of 1-tetradecene and cembrene increased in AQ2 and AQ3.

**Fig. 3:**
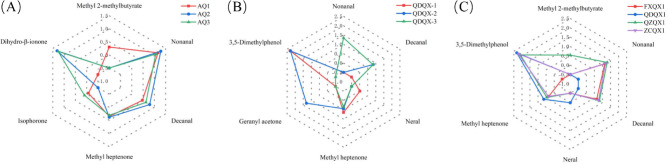
OAV flavor profile analysis of cigar tobacco leaves. The OAV profile was expressed as the log of OAV from main volatiles. (**A**) Wrapper of different varieties. (**B**) Filler of different varieties. (**C**) Filler of different origins.

As shown in Fig. 3A, the flavor constituents of the first six high OAVs in the three wrapper varieties of CTLs included methyl 2-methylbutyrate, nonanal, decanal, methyl heptenone, isophorone, and dihydro-β-ionone. Among them, the OAVs of nonanal, decanal, methyl heptenone, and dihydro-β-ionone were the highest in AQ2, which imparted strong citrus, orange peel, and floral aromas. The OAVs of methyl 2-methylbutyrate and isophorone were the highest in AQ1 and AQ3, giving apple and cedarwood aromas, respectively.

### Effect of variety on aroma profiles and differential constituents of filler CTLs

There were 63 aroma constituents detected in filler CTLs of three varieties, including esters (8), alcohols (13), acids (1), aldehydes (10), ketones (16), phenols (1), nitrogen heterocycles (10), and alkenes (4), and their total contents were 2.72–4.27 mg/kg (Table S2). Each of these components has a different concentration, which gave cigar its unique olfactory properties and distinguished it from other types of tobacco^31^. There were also significant differences in the varieties and contents of aroma constituents. Among them, the contents of ketones, alkenes, and total aroma constituents of QDQX2 increased, the contents of esters, alcohols, and aldehydes of QDQX3 increased, and the contents of nitrogen heterocycles increased both in QDQX2 and QDQX3 (Fig. 1B). There were significant differences in the proportion of aroma profiles between different filler varieties for esters (2.36–7.46%), alcohols (3.46–11.62%), aldehydes (1.24–3.42%), ketones (22.34–47.56%), and alkenes (1.23–5.16%). The filler, which occupied a central position in a cigar, was prioritized as the main ingredient, and it usually accounted for 70% to 85% of the total weight of the cigar, which has a significant impact on the style and characteristics of the cigar^32^.

The total esters content of QDQX3 increased significantly, reaching 241.01 μg/kg, due to the increased contents of dihydroactinidiolide and methyl butanoate. Acetoxyethane was newly detected and the contents of lavender lactone and norambreinolid increased in QDQX1. The total alcohols content of QDQX3 reached 375.09 μg/kg, which was 56.59% and 153.98% higher than QDQX1 and QDQX2, respectively, as the newly detected 3-acetopropanol and methylundecylcarbinol (Fig. 2B), and the contents of 4,8-dimethylnonanol, phytol, hexa-hydro-farnesol, thunbergol, and geranylgeraniol were significantly increased. Dodecanol and isophytol were newly found in QDQX2. The total aldehydes content of QDQX3 increased by 83.93% and 109.38% compared with QDQX1 and QDQX2, respectively, due to the newly detected nonanal and the increase in octanal content. Neral was newly detected and the contents of methacrylaldehyde, valeraldehyde, prenal, hexanal, benzaldehyde, and 3-methylpentanoic acid increased significantly in QDQX1.

The total ketones content of QDQX2 reached 2.03 mg/kg, which was 1.50 and 1.81 times higher than that of QDQX1 and QDQX3, respectively, due to the newly detected geranyl acetone and cis-ψ-ionone, whose contents were 1.21 mg/kg and 83.19 μg/kg, respectively, and the contents of pseudoionone and farnesyl acetone increased significantly. Methyl tridecyl ketone was newly detected, and the contents of acetone, butenone, methyl heptenone, 5-ethyl-6-methyl-3(E)-hepten-2-one, and ketoisophorone were increased in QDQX1, while isophorone, solanone, and megastigmatrienone were newly detected in QDQX3. As tobacco was processed and aged, solanone underwent a further transformation to produce compounds such as somnirol, solanofuran, and norsolandione, and these derivatives have a considerable effect on the taste of tobacco products^33^. Besides, dihydrooxophorone and 4,8-dimethylnona-3,7-dien-2-one were detected, and the content of hexahydropseudoionone increased in QDQX1 and QDQX2.

The total nitrogen heterocycles content of QDQX3 was significantly higher than that of QDQX1, due to the newly detected nicotyrine in QDQX3, with the content reaching 637.07 μg/kg. Pyridine, methylpyrazine, and 3-methylpyrrole were newly detected, and the content of 2,5-dimethylpyrrole increased in QDQX2. The contents of 2-methylpyrrole and nicotinotrile in QDQX1 increased, and the contents of myosmine and isonicoteine in QDQX1 and QDQX2 increased significantly. The total alkenes content of QDQX2 increased significantly, reaching 219.98 μg/kg, as 1-nonadecene was detected, and its content was 193.75 μg/kg. Amorphadiene and cembrene were detected in QDQX1 and QDQX3, respectively, while the 1-acetylcyclohexene content of QDQX1 and QDQX2 increased significantly.

As shown in the Fig. 3B, the flavor constituent of the first six high OAVs in the three filler varieties of CTLs included nonanal, decanal, neral, methyl heptenone, geranyl acetone, and 3,5-dimethylphenol. Among them, the OAVs of neral, methyl heptenone, and 3,5-dimethylphenol were the highest in QDQX1, which imparted strong lemon, citrus, and balsamic aromas. The OAVs of decanal and geranyl acetone were the highest in QDQX2, and orange peel and fruity aromas were prominent.

### Effect of origin on the aroma profiles and differential constituents of filler CTLs

A total of 64 aroma constituents were detected in four filler origins of CTLs, including esters (9), alcohols (10), acids (1), aldehydes (11), ketones (18), phenols (1), nitrogen heterocycles (9), and alkenes (5), and their total content was ranged from 1.74 mg/kg to 2.87 mg/kg (Table S3). There were significant differences in the varieties and contents of aroma constituents, which showed the origin specificity. Among them, the contents of esters, ketones, nitrogen heterocycles, alkenes, and total aroma constituents of FXQX1 increased, while the contents of alcohols and aldehydes of QDQX1 and QZQX1 increased (Fig. 1C). Besides, there was a significant difference in alcohol proportion (2.06–11.88%) between filler CTLs from different origins. Ketones and nitrogen heterocycles were dominant, with the proportions of 38.66–44.72% and 36.74–43.63%, respectively, the esters, aldehydes, and alkenes were 3.17–11.74%, 2.13–4.90%, and 0.39–2.80%, respectively. The aroma precursor varieties and contents of CTLs from different origins were significantly different, which showed the obvious origin specificity, while different origins in proximity had high similarity in their aroma profiles^34^.

The total esters content of FXQX1 and ZCQX1 increased significantly, due to the increase in dihydroactinidiolide content, which were 182.36 μg/kg and 181.47 μg/kg, respectively. Ethyl acetate and farnesyl acetate were newly detected (Fig. 2C), and the contents of lavender lactone and sclareolide were increased in QDQX1. Methyl carbonate, methyl propylate, and methyl 2-methylbutyrate were newly detected, and the content of methyl butanoate increased in QZQX1.

The total alcohols content of QDQX1 reached 239.54 μg/kg, which was 1.67–4.33 times higher than that of other origins, due to the newly detected nerolidol, hexa-hydro-farnesol, and 2-hexyldecanol, and the contents of 6,10,14-trimethylpentadecan-2-ol and thunbergol significantly increased. Tetradecanol, isophytol, and phytol were detected, and the content of 3,7,11,15-tetramethyl-2-hexadecen-1-ol increased in FXQX1. The total aldehydes content of QZQX1 increased by 32.76–75.68% compared with other origins, as the newly detected octanal and pentadecanal, and the contents of trans-2-hexenal, nonanal, and decanal increased significantly. Methacrylaldehyde and pentanal were newly detected, and the contents of prenal, hexanal, and benzaldehyde increased in QDQX1.

The total ketones content of FXQX1 increased significantly, reaching 1.24 mg/kg, which increased by 28.70–84.14% compared with other origins, due to the significant increase in the contents of geranyl acetone and megastigmatrienone, reaching 856.40 μg/kg and 124.01 μg/kg, respectively, and the newly detected isophorone and cis-ψ-ionone. Isophorone, a degradation by-product of carotenoids, belonged to the terpenoid family and was known for its powerful and long-lasting aroma, which significantly enhanced the flavor of different tobacco types^35^. Acetone and 5-ethyl-6-methyl-3(E)-hepten-2-one were newly detected, and the contents of butenone, methyl heptenone, ketoisophorone, dihydrooxophorone, 4,8-dimethylnona-3,7-dien-2-one, hexahydropseudoionone, methyl tridecyl ketone, farnesyl acetone, and 3,5-dimethylphenol increased in QDQX1, and hexahydropseudoionone and farnesyl acetone reached 260.63 μg/kg and 358.53 μg/kg, respectively. 5,6-Epoxy-β-ionone was newly found, and the contents of pseudoionone, solavetivone, and 3-methylpentanoic acid increased in ZCQX1.

The total nitrogen heterocycles content of FXQX1 reached 1.25 mg/kg, which increased by 43.82–96.45% compared with other origins, due to methylpyrrolidone being newly detected, and the nicotyrine content increased significantly, reaching 884.36 μg/kg. The contents of 2-methylpyrrole, myosmine, and isonicoteine in QDQX1 were also significantly increased, reaching 57.43 μg/kg, 233.41 μg/kg, and 402.42 μg/kg, respectively. Pyridine was newly detected, and the content of 1-methylpyrrole increased in QZQX1. The total alkenes content of FXQX1 increased significantly, as the newly detected bicylogermacrene and cembrene, and amorpha-4, 11-diene and β-ocimene were detected in QDQX1 and QZQX1, respectively.

The flavor constituents of the first six high OAVs included methyl 2-methylbutyrate, nonanal, decanal, neral, methyl heptenone, and 3,5-dimethylphenol (Fig. 3C). Among them, the OAVs of neral, methyl heptenone, and 3,5-dimethylphenol were the highest in QDQX1, giving it strong lemon, citrus, and balsamic aromas. The OAVs of methyl 2-methylbutyrate, nonanal, and decanal were the highest in QZQX1, with apple, citrus, and orange peel aromas.

### Effect of variety on the microbial community diversity and structure of wrapper CTLs

The high-throughput sequencing technology was used to explore the microbial community characteristics of CTLs from different varieties and origins. The effective sequence range of bacteria and fungi in all samples were 62,338–83,465 and 19,537–98,236, respectively. As the sequencing depth increased, the dilution curve gradually became flat, indicating that the sequencing results were sufficient to reflect the diversity of samples.

As shown in Tables 1 and 2, for the bacterial community, Chao1 and Observed_OTUs indices were higher in AQ2, thereby the community richness was higher. The Shannon and Pielou_e indices and community diversity and evenness were high in AQ3, while the community richness, Shannon, and Pielou_e indices were lower in AQ1. For the fungal community, the community richness, diversity, and evenness were the highest in AQ1, while they were the lowest in AQ2.

**Table 1:** The bacterial community α-diversity indices of cigar tobacco leaves from different wrapper and filler varieties and filler origins.

Sample	Chao 1	Observed OTUs	Shannon	Simpson	Pielou_e	Dominance
AQ1	265.250	264	2.950	0.774	0.367	0.226
AQ2	326.167	326	3.223	0.766	0.386	0.234
AQ3	311.000	311	3.474	0.762	0.419	0.238
QDQX1	479.545	479	3.927	0.797	0.441	0.203
QDQX2	351.429	351	3.418	0.792	0.404	0.208
QDQX3	202.364	201	2.753	0.756	0.360	0.244
QZQX1	84.000	84	3.391	0.773	0.531	0.227
ZCQX1	171.000	171	1.640	0.415	0.221	0.585
FXQX1	501.000	501	6.027	0.895	0.672	0.105

**Table 2:** The fungal community α-diversity indices of cigar tobacco leaves from different wrapper and filler varieties and filler origins.

Sample	Chao 1	Observed OTUs	Shannon	Simpson	Pielou-e	Dominance
AQ1	395.568	371	4.724	0.892	0.553	0.108
AQ2	217.559	208	2.203	0.558	0.286	0.442
AQ3	230.452	223	3.934	0.835	0.504	0.165
QDQX1	366.000	366	5.258	0.928	0.617	0.072
QDQX2	296.022	293	4.750	0.891	0.580	0.109
QDQX3	268.200	255	2.713	0.590	0.339	0.410
QZQX1	399.600	373	3.526	0.748	0.413	0.252
ZCQX1	349.038	345	4.888	0.910	0.580	0.090
FXQX1	228.667	225	4.183	0.867	0.535	0.133

**Fig. 4:**
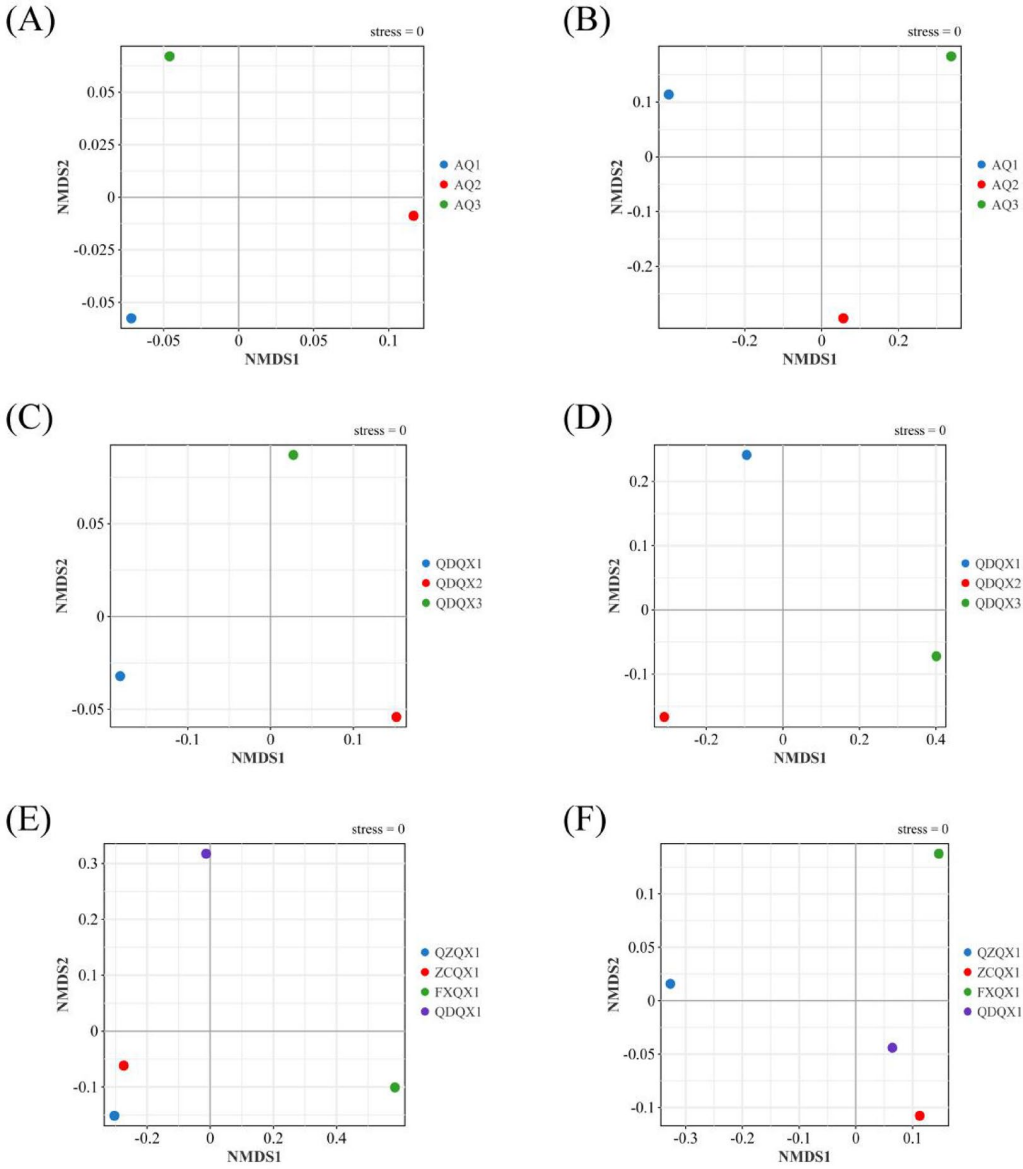
Non-metric multidimensional scaling (NMDS) analysis of the microbial community of cigar tobacco leaves at the genus level (OTU > 1%). (**A**) Bacterial community of different wrapper varieties. (**B**) Fungal community of different wrapper varieties. (**C**) Bacterial community of different filler varieties. (**D**) Fungal community of different filler varieties. (**E**) Bacterial community of different filler origins. (**F**) Fungal community of different filler origins.

NMDS analysis showed that there were obvious differences in the community structure of different varieties of wrapper CTLs (Fig. 4A,B). For the bacterial community, *Corynebacterium* and *Staphylococcus* were dominant in the three varieties, but their abundances were different. For the fungal community, the abundances of *Alternaria*, *Nigrospora*, and *Cladosporiu*m were higher in AQ1. *Alternaria* was dominant, and *Aspergillus* abundance was higher in AQ2, while *Aspergillus* was dominant in AQ3.

**Fig. 5:**
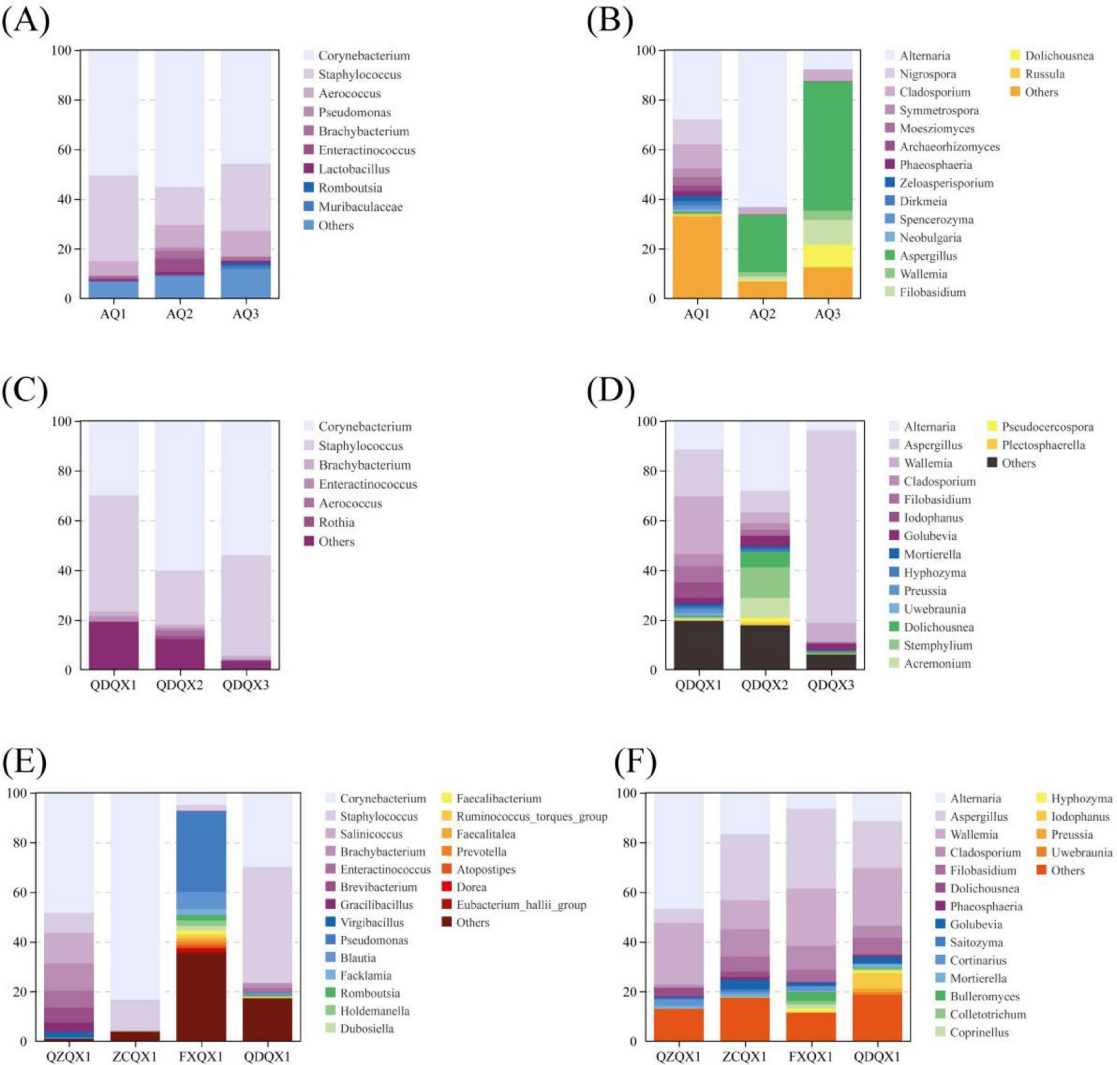
The microbial community composition of cigar tobacco leaves at the genus level (OTU > 1%). (**A**) Bacterial community of different wrapper varieties. (**B**) Fungal community of different wrapper varieties. (**C**) Bacterial community of different filler varieties. (**D**) Fungal community of different filler varieties. (**E**) Bacterial community of different filler origins. (**F**) Fungal community of different filler origins.

The bacterial communities of different varieties of wrapper CTLs included 23 phyla, 43 classes, 104 orders, 156 families, and 915 genera. The dominant bacterial genus composition and abundance of the three varieties of CTLs were slightly different at the genus level (Fig. 5A). The dominant bacteria were *Corynebacterium* and *Staphylococcus*, which was consistent with other finding^36^, with abundances of 45.78–55.34% and 15.16–34.60%, respectively, and the total abundances of the two and *Aerococcus* were 79.50–90.89%. The abundances of *Brachybacterium* and *Enteractinococcus* in AQ2 increased slightly. When cigars undergo stack fermentation, *Staphylococcus* and *Aerococcus* could utilize sugars and acids in the CTLs, leading to an increase in stack temperature and pH, which in turn promoted the growth of *Corynebacterium*^37^. These three strains exhibited stronger resistance to alkali and salt, giving them a competitive advantage during the stacking fermentation process^37^. *Staphylococcus* played a crucial role in carbohydrate catabolism, amino acid conversion, protein hydrolysis, and lipolysis. It mediated the breakdown of branched-chain amino acids into methyl-branched-chain alcohols, aldehydes, carboxylic acids, and their corresponding esters, establishing itself as a key genus in flavor development^38^. It could quickly metabolize malic acid and citric acids in tobacco, and these organic acids influenced the smoking quality of the tobacco^37^. *Corynebacterium* produced aldehydes and ketones, which were crucial in enhancing the flavor profile of cigars^39^.

The fungal communities included 8 phyla, 27 classes, 66 orders, 135 families, and 194 genera. The composition and abundance of the fungal communities were significantly different (Fig. 5B). Among them, the abundances of *Alternaria*, *Nigrospora*, and *Cladosporium* in AQ1 were higher, with abundances of 27.95%, 10.09%, and 9.76%, respectively. *Alternaria* was dominant and *Aspergillus* was higher in AQ2, with abundances of 63.24% and 23.01%, respectively. *Aspergillus* was dominant with an abundance of 51.90%, while the abundances of *Filobasidium*, *Dolichousnea*, and *Alternaria* increased to 9.98%, 9.23%, and 7.85% in AQ3, respectively. The role of fungi in modulating CTLs may be associated with the degradation of lignin, cellulose, and pectin^40^. *Alternaria* was capable of degrading cellulose in CTLs, which contributed to the biosynthesis of sugar derivatives^41^. *Aspergillus* promoted the degradation of sugars and proteins as well as the synthesis of aroma compounds in tobacco, which capable of producing various enzymes such as proteases and amylases, to enhance the production of aromatic compounds, to convert carbohydrates into organic acids, alcohols, and esters, thus improving the quality of cigars. *Cladosporium* was a key filamentous fungus that produced amylase, protease, and cellulase enzymes, which were responsible for breaking down macromolecules and producing characteristic flavor components^42^.

### Effect of variety on the microbial community diversity and structure of filler CTLs

Based on the α-diversity indices analysis, the richness, diversity, and evenness were the highest in QDQX1 both for the bacterial and fungal communities, while they were the lowest in QDQX3. According to the NMDS analysis (Fig. 4C,D), there were obvious differences in the bacterial and fungal community structures of different filler varieties of CTLs. Among them, *Corynebacterium* and *Staphylococcus* were dominant in the bacterial communities, but there were significant differences in their abundances. For fungal communities, the abundances of *Wallemia*, *Aspergillus*, and *Alternaria* were higher in QDQX1, and the abundances of *Alternaria* and *Stemphylium* were higher in QDQX2, while *Aspergillus* was dominant in QDQX3.

The bacterial communities of different filler varieties of CTLs included 25 phyla, 40 classes, 99 orders, 148 families, and 228 genera. The compositions of dominant bacterial genera were similar, but there were obvious differences in abundance at the genus level (Fig. 5C). The dominant bacteria were *Corynebacterium* and *Staphylococcus*, and their total abundances were 76.67–94.47%, but the abundances of *Corynebacterium* and *Staphylococcus* in QDQX1 were 29.97% and 46.70%, they were 60.90% and 22.12% in QDQX2, while they were 54.04% and 40.43% in QDQX3, respectively.

The fungal communities included 10 phyla, 28 classes, 61 orders, 121 families, and 179 genera. There were significant differences in the composition and abundance of the fungal community (Fig. 5D). For example, the abundances of *Wallemia*, *Aspergillus*, and *Alternaria* were higher in QDQX1, with abundances of 23.20%, 18.84%, and 11.50%, respectively. The abundances of *Alternaria*, *Stemphylium*, *Aspergillus*, and *Acremonium* were higher in QDQX2, with abundances of 28.17%, 12.47%, 8.74%, and 7.89%, respectively. *Aspergillus* was dominant in QDQX3, with an abundance of 77.17%. The dominant microorganisms possess the ability to degrade, transform, metabolize, and utilize the matrix components of CTLs, thereby playing a vital role in shaping their quality characteristics^43^. The secretion of α-amylase and amylase by *Staphylococcus* and *Aspergillus* was the primary cause of the changes in starch and total sugar content^44^.

Varieties have little effect on the bacterial community structures of wrapper and filler varieties of CTLs, and there were only differences in their abundances, but there were obvious differences in fungal community composition and abundance. For example, in the fermented wrapper, binder, and filler CTLs, the bacterial community structures were more similar compared to the fungal microbiota, and seven fungal genera were identified as functional microorganisms that may play a crucial role in maintaining the sustainability and stability of the phyllosphere ecosystem^19^. The wrapper CTLs contained a greater diversity of characteristic fungal genera compared to the filler and underwent fermentation more rapidly, which suggested that the specifics of the fermentation process should be appropriately adjusted to ensure consistent quality when dealing with plant leaves of different genotypes^45^. The dominant microbes during the cigar stacking fermentation of eight varieties of CTLs primarily influenced the microbial community structure and characteristic microorganisms through microbial interactions, thereby affecting the transformation of volatile flavor compounds^6^. However, the dominant bacterial and fungal genera of different CTL varieties during fermentation were similar^6^, which indicated that the fungal communities were more sensitive to environmental conditions than bacteria after fermentation.

The distinctive flavor of CTLs was largely attributed to the metabolic activities of the microbial community composition, which included the degradation of carbohydrates and chlorogenic acid, the breakdown of proteins, the biosynthesis of fatty acids and lipids, and the biosynthesis of amino acids and aromatic compounds^37,46^. The main pathways used by bacteria to break down macromolecules during CTL fermentation included carbohydrate metabolism, lipid metabolism, and amino acid metabolism^16^. Intermediates from carbohydrate metabolic pathways could act as raw materials for synthesizing a variety of substances. Amino acids could participate in the Maillard reaction with reducing sugars, a crucial reaction that contributed to the development of aroma in tobacco^47^. The microbial-mediated hydrolysis of peptides and/or proteins, along with the advancement of the Maillard reaction, played a significant role in modifying the distribution of free amino acids, thereby shaping the distinct flavor profile of CTLs^48^. The methylerythritol phosphate pathway and the carotenoid synthesis pathway were key contributors to flavor development in CTL from different origins^36^.

### Effect of origin on the microbial community diversity and structure of filler CTLs

The richness, diversity, and evenness of FXQX1 were the highest in the bacterial community, while the richness of QZQX1 and the diversity and evenness of ZCQX1 were the lowest. For the fungal community, the richness of QZQX1 and the diversity and evenness of QDQX1 were the highest, while the richness of FXQX1 and the diversity and evenness of QZQX1 were the lowest. Based on the NMDS analysis (Fig. 4E,F), the bacterial community structures of QZQX1 and ZCQX1 were similar, and there were obvious differences between the two and FXQX1 or QDQX1. Among them, *Corynebacterium* was dominant and *Staphylococcus* abundance was higher in the communities of QZQX1 and ZCQX1, while *Pseudomonas* was dominant in FXQX1, *Staphylococcus* was dominant and *Corynebacterium* abundance was higher in QDQX1. Besides, the fungal community structures of ZCQX1 and QDQX1 were similar, and there were obvious differences between the two and QZQX1 or FXQX1. The abundances of *Aspergillus*, *Wallemia*, and *Alternaria* were higher in communities of ZCQX1 and QDQX1, while the abundances of *Alternaria* and *Wallemia* were higher in QZQX1, and the abundances of *Aspergillus* and *Wallemia* were higher in FXQX1.

The diversity and dynamics of microbial communities were closely related to environmental factors such as climate, soil composition, and agricultural practices, and varied across tobacco-growing regions. The bacterial communities of filler CTLs from different origins included 23 phyla, 37 classes, 73 orders, 166 families, and 275 genera. There were significant differences in the composition and abundance of bacterial communities at the genus level (Fig. 5E). The *Corynebacterium* abundance was higher in QZQX1 and ZCQX1, the former was 48.42% and the latter was 83.25%, the abundances of *Salinicoccus*, *Brachybacterium*, *Enteractinococcus*, and *Brevibacterium* in QZQX1 were also higher, with abundances of 12.28%, 11.01%, 6.72%, and 6.10%, respectively. *Staphylococcus* was dominant, and *Corynebacterium* abundance was higher in QDQX1, with abundances of 46.70% and 29.97%, respectively. The abundances of *Pseudomonas*, *Blautia*, and *Corynebacterium* were higher in FXQX1, with abundances of 32.48%, 7.06%, and 5.02%, respectively. *Pseudomonas* promoted the enzymatic degradation of proteins, enriching the pool of amino acids, which significantly contributed to flavor development. Some strains were also capable of degrading nicotine in tobacco, helping to adjust tobacco strength and improve the quality of upper tobacco leaves^49,50^.

The fungal communities included 12 phyla, 36 classes, 278 orders, 143 families, and 226 genera. The dominant fungal genus composition was similar, but the abundance was different (Fig. 5F). *Alternaria*, *Aspergillus*, *Wallemia*, and *Cladosporium* were dominant in filler CTLs from different origins, and their total abundances were 58.43–78.41%. Among them, the abundances of *Alternaria* and *Wallemia* were higher in QZQX1, which were 46.73% and 24.84%, respectively. The abundances of *Aspergillus*, *Alternaria*, *Wallemia*, and *Cladosporium* in ZCQX1 were higher, with abundances of 26.63%, 16.68%, 11.80%, and 10.89%, respectively. The abundances of *Aspergillus*, *Wallemia*, and *Cladosporium* in FXQX1 were higher, with abundances of 32.24%, 23.07%, and 9.54%, respectively. The abundances of *Wallemia*, *Aspergillus*, and *Alternaria* in QDQX1 were higher, with abundances of 23.20%, 18.84%, and 11.50%, respectively. *Pseudomonas* spp., *Aspergillus fumigatus*, and *Rhodococcus* spp. degraded nicotine in tobacco into niacin, ethanesulfonic acid, oxidized nicotine, nicotinamide, N-methylnicotinamide, and adamantane^51^.

Microbial communities from different origins differed significantly from each other, with bacterial microorganisms being more diverse and abundant than fungi, which was in agreement with other study^36^. It was the bacterial community, rather than the fungal community, that played a key role in differentiating cigars from the Americas, Southeast Asia, and East Asia, which in turn influenced microbial metabolism^52^. For CTLs from Dominica, Brazil, Indonesia, and China, the relationships between the fungal community and volatile flavor compounds were weaker compared to those with the bacterial community, and most of these relationships were negative, indicating that the bacterial community had a greater impact on CTL flavor^12^. However, for CTL sourced from six regions of Yunnan Province in China, the influence of fungal microflora on metabolites was more pronounced. In this case, fungi exhibited a stronger regional impact on microbial communities than bacteria, likely due to the shorter geographical distances between the CTLs compared to other studies.

The microbial community on CTLs primarily originated from the soil and surrounding environment during field growth, as well as from the air during the drying process. Geographic factors were the dominant influence shaping the composition of the tobacco microbial community, and differences in geospatial locations led to variations in microbial community structure^53^. Through intricate interactions, microorganisms from various origins collaborate to influence the structure and function of the entire microbial community, thereby impacting the flavor quality of CTLs. Changes in community structure and composition were largely associated with shifts in filler tobacco leaves, which stemmed from alterations in their metabolic activities^54^. Besides, geographical location primarily influenced the structure of phyllosphere microbial communities through the following mechanisms: (1) Climate factors, climatic variations, soil properties, and phyllosphere functional traits generally varied with geographical location. (2) The spatial distribution patterns of contemporary natural vegetation were linked to geographical location. (3) Historical changes in elevation have reshaped the spatial distribution of climate variables and vegetation^55,56^. Plant species or varieties influenced the structure of the phyllosphere microbial community primarily through the following mechanisms: (1) Differences in the assembly mechanisms of the phyllosphere microbial community, ecological strategies, leaf morphological structures, and physicochemical properties among various plant species or varieties^57^. (2) Parental “vertical transmission” served as a significant source of phyllosphere microorganisms, and the capacity for this vertical transmission may vary among different plant species or varieties. (3) In the case of phyllosphere microbial communities associated with trees or perennial species, the migration of microorganisms from the soil to the phyllosphere may be less frequent due to greater physical distance and the extended time required for host adaptation^58^.

Microbial recruitment in the phyllosphere appeared to be evolutionarily complex and was thought to occur based on the functional significance of different microbial communities to plant traits and the ecological strategies of the host plant. Temporal or seasonal variations bring about significant changes in temperature, humidity, and solar radiation levels, which in turn induce corresponding shifts in the structure and composition of the foliar microflora^59^. The seasonality and dynamics of bacterial and fungal communities in the phyllosphere have been effectively predicted^60^. It was now widely recognized that short-term variations in the microbial communities of the phyllosphere were driven by stochastic weather events, such as strong winds and heavy rainfall, which directly influenced the selective growth and colonization of microbes on leaf surfaces^61^. The long-term seasonal changes in these communities were attributed to variations in seasonal climate, microbial succession, and the age of the plant host^62^. The predicted biogeography was a key factor distinguishing foliar microbial communities, even among individuals of the same plant species. This distance-decay relationship suggested that plants growing closer together have more similar phyllosphere microbial communities, with this similarity decreasing as geographic distance increased^63^.

### The correlation between aroma constituents and dominant microorganisms

**Fig. 6:**
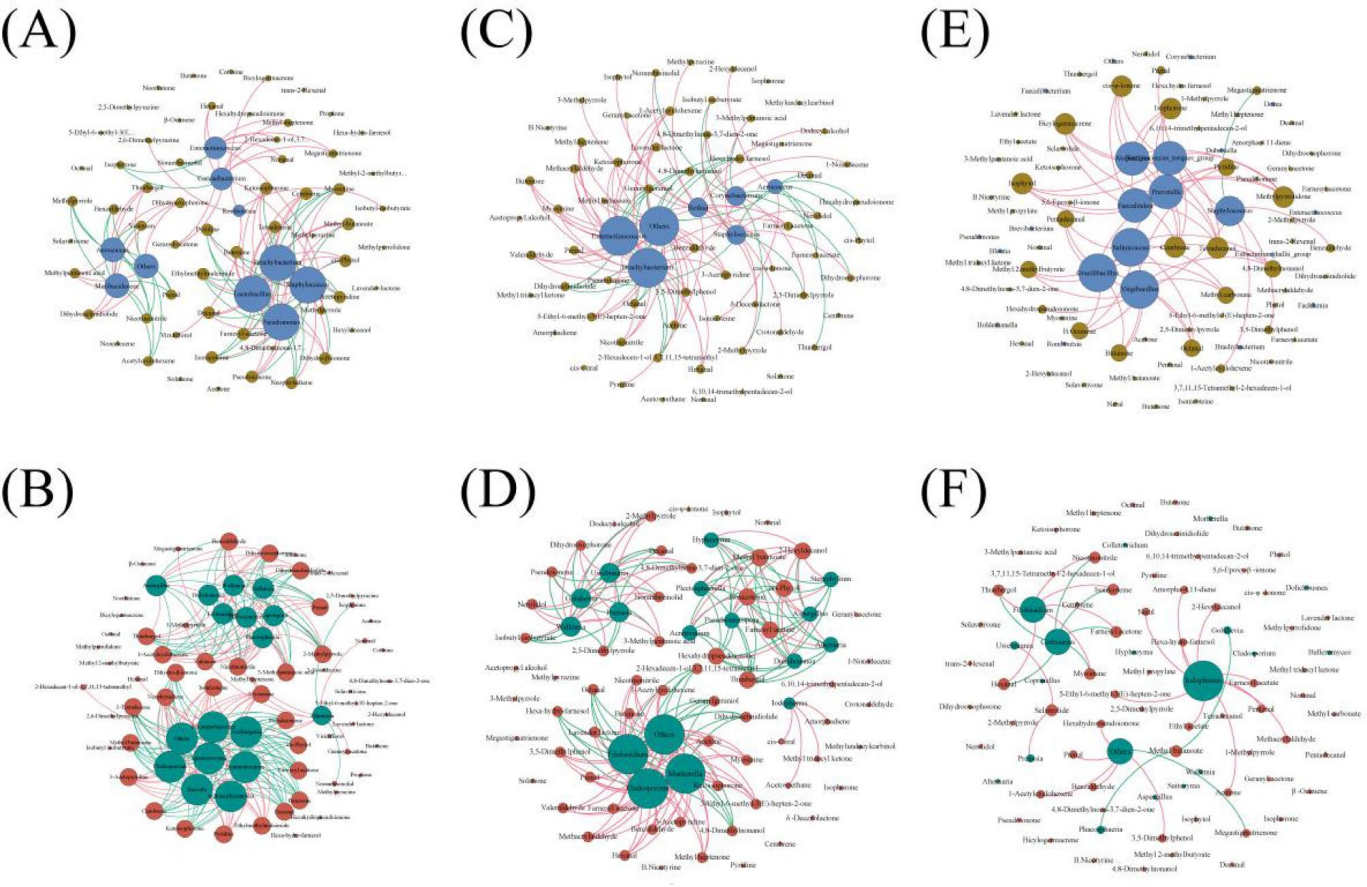
Correlation analysis of aroma constituents and dominant microbes (OTU > 1%, *P* < 0.05, and Spearman’s |RHO|> 0.6). (**A**) Bacterial community of different wrapper varieties. (**B**) Fungal community of different wrapper varieties. (**C**) Bacterial community of different filler varieties. (**D**) Fungal community of different filler varieties. (**E**) Bacterial community of different filler origins. (**F**) Fungal community of different filler origins.

As shown by correlation analysis (Fig. 6A), *Romboutsia* in the bacterial community of different wrapper varieties of CTLs was strongly correlated (*P* < 0.001) with the aroma constituents of isobutyl isobutyrate, 2,6-dimethylpyrazine, viridiflorol, and bicylogermacrene. *Lactobacillus* was associated (*P* < 0.05) with decanal and farnesyl acetone. Fungal communities of *Nigrospora*, *Dirkmeia*, *Moesziomyces*, *Phaeosphaeria*, *Archaeorhizomyces*, *Zeloasperisporium*, *Russula*, *Spencerozyma*, *Neobulgaria*, and *Symmetrospora* were correlated (*P* < 0.01) with methyl 2-methylbutyrate, hexa-hydro-farnesol, octanal, acetone, butanone, propione, solanone, nootkatone, solavetivone, methylpyrazine, 2,5-dimethylpyrazine, methylpyrrolidone, and cotinine (Fig. 6B).

*Rothia* and *Pseudocercospora* in the microbial communities of different filler varieties of CTLs were closely related (*P* < 0.001) to the aroma constituents of δ-decenolactone, dodecyl alcohol, isophytol, crotonaldehyde, geranyl acetone, cis-ψ-ionone, pyridine, methylpyrazine, 3-methylpyrrole, and 1-nonadecene (Fig. 6C,D), and *Brachybacterium* (*P* < 0.01) and *Iodophanus* (*P* < 0.001) were associated with acetoxyethane, 6,10,14-trimethylpentadecan-2-ol, cis-citral, methyl tridecyl ketone, and amorphadiene. *Corynebacterium* in the bacterial community was associated (*P* < 0.05) with isobutyl isobutyrate, pseudoionone, and 2,5-dimethylpyrrole. *Filobasidium* in the fungal community was correlated (*P* < 0.05) with lavender lactone, 5-ethyl-6-methyl-3(E)-hepten-2-one, nicotinonitrile, prenal, and ketoisophorone.

*Alternaria* (*P* < 0.05), *Salinicoccus*, *Gracilibacillus*, *Virgibacillus,* and *Brevibacterium* in the microbial communities from different filler origins of CTLs were closely related (*P* < 0.001) to the aroma constituents of tetradecanol, isophytol, isophorone, cis-ψ-ionone, methylpyrrolidone, bicylogermacrene, and cembrene (Fig. 6E,F). Bacterial communities of *Pseudomonas*, *Ruminococcus_torques_group*, *Faecalitalea*, *Prevotella*, *Atopostipes, Facklamia,* and *Dorea* were closely associated (*P* < 0.001) with methyl carbonate, methyl 2-methylbutyrate, 4,8-dimethylnonanol, octanal, pentadecanal, butanone, pyridine, β-ocimene. Fungal communities of *Iodophanus* and *Uwebraunia* were closely associated (*P* < 0.001) with 5,6-epoxy-β-ionone and 2,5-dimethylpyrrole. *Bulleromyces* and *Coprinellus* were closely associated (*P* < 0.01) with methyl carbonate, methyl 2-methylbutyrate, 4,8-dimethylnonanol, octanal, pentadecanal, butanone, pyridine, and β-ocimene, whereas *Phaeosphaeria* was associated (*P* < 0.05) with prenal, hexanal, myosmine, butenone, farnesyl acetone, and 1-acetylcyclohexene.

Correlation analysis has facilitated the screening of functional microorganisms and the development and application of microbial agents aimed at enhancing flavor quality and producing characteristic aromatic cigars. For example, in vitro isolation and bioaugmentation inoculation with *Candida parapsilosis* and *Candida metapsilosis* have been shown to significantly decreased alkaloid content while increasing the concentration of flavor components in tobacco leaves^64^. The addition of *Bacillus altitudinis* enhanced the transformation of macromolecules in CTLs and effectively promoted aroma production, the total aroma production increased by 43% compared to natural fermentation^65^. The contents of solanone, 6-methyl-5-hepten-2-one, benzeneacetic acid ethyl ester, cyclohexanone, octanal, acetophenone, and 3,5,5-trimethyl-2-cyclohexen-1-one in CTLs were significantly elevated following the inoculation with *Acinetobacter* sp. 1H8^66^. The changes in microbial communities were primarily associated with their varied functions during fermentation. This suggested that when the fermentation effect of the original microbial community in CTLs was not optimal, we could optimize or design the microbial community based on the specific fermentation functions needed^67^. Besides, the correlation analyses have shown that microorganisms affect tobacco aroma through synergistic effects, therefore, when searching for functional microorganisms to improve the aroma quality of tobacco, it was important to consider the synergistic effects between microorganisms^68^.

## Conclusion

Aroma constituents of the ketones and nitrogen heterocycles were dominant in wrapper and filler CTLs from different varieties and filler CTLs from different origins. The proportions of the two in different CTL varieties varied greatly, while the differences between various origins were not obvious. There were significant differences in the varieties and contents of aroma constituents in CTLs of different varieties and origins, which exhibited obvious variety and origin specificity. For example, the contents of alcohols, nitrogen heterocycles, and total aroma constituents in the wrapper variety of AQ1 increased significantly, while the contents of aldehydes, ketones, and alkenes of AQ2 increased. The contents of ketones, alkenes, and total aroma constituents in the filler variety of QDQX2 increased significantly, while the contents of esters, alcohols, and aldehydes of QDQX3 increased. The contents of ketones, nitrogen heterocycles, alkenes, and total aroma constituents in the origin of FXQX1 increased significantly, while the alcohols and aldehydes in QDQX1 and QZQX1 increased, respectively. Besides, the microbial community diversity and structure of CTLs were significantly affected by variety and origin. There were obvious differences in the α- and β-diversity of microbial communities in different varieties and origins. Varieties have little effect on the bacterial communities of wrapper and filler varieties of CTLs, *Corynebacterium* and *Staphylococcus* were dominant and there were only differences in their abundances, but there were obvious differences in fungal community composition and abundance. Different from the influence of variety, the composition and abundance of the bacterial community were significantly changed by the origin. *Alternaria*, *Aspergillus*, *Wallemia*, and *Cladosporium* were dominant in the fungal communities, with only differences in their abundances. The correlation analysis showed that the formation of aroma profiles of CTLs from different wrapper and filler varieties and filler origins was closely related to the microbial communities, with the synergistic effect of different bacterial and fungal communities, and the microbial communities that promoted the formation of the aroma profiles were obviously different among different wrapper and filler varieties and filler origins.

## Materials and methods

### Materials and reagents

The chemical reagents utilized in the experiments were procured from Sinopharm Chemical Reagent Co., Ltd (Shanghai, China). The phenethyl acetate standard was obtained from Anpel Laboratory Technologies Inc. (Shanghai, China). The CTLs were harvested in 2021 and obtained from the production regions in Shandong, China (Table 3).

**Table 3:** The cigar tobacco leaves used in this study.

No	Name	Origin	Variety
1	AQ1	Anqiu	Qingyi1
2	AQ2	Anqiu	Qingyi2
3	AQ3	Anqiu	Qingyi3
4	QDQX1	Qingdao	Qingxin1
5	QDQX2	Qingdao	Qingxin2
6	QDQX3	Qingdao	Qingxin3
7	FXQX1	Feixian	Qingxin1
8	QZQX1	Qingzhou	Qingxin1
9	ZCQX1	Zhucheng	Qingxin1

### Analysis of aroma constituents

The aroma constituents of CTLs were analyzed by HS–SPME–GC–MS, in accordance with the method of the reference^29^. In summary, 0.5 g of CTL powder was weighed and placed in a 20 mL headspace vial. Subsequently, 2 μL of 105 mg/L phenethyl acetate was added, the vial was rapidly sealed and shaken, and the mixture was heated at 70 °C for 30 min. Then the mixture was extracted using a 50/30 μm DVB/CAR/PDMS fiber (Supelco Inc., Bellefonte, PA, USA) for 30 min.

The analysis was conducted using a GC–MS (7890A/5977C, Agilent Technologies Inc., Santa Clara, CA, USA) equipped with an elastomeric quartz capillary column (DB-5MS, 30 m × 0.25 mm × 0.25 μm, Agilent Technologies Inc.). The heating program was set to an initial temperature of 40 °C and maintained for 2 min. Thereafter, the temperature was increased to 220 °C at a rate of 6 °C/min and maintained for 0 min. Subsequently, the temperature was elevated to 280 °C at a rate of 20 °C/min and maintained for 10 min. The temperature of the inlet was 240 °C, and the carrier gas was helium (99.999%). The conditions of mass spectrometry were as follows: electron impact (EI) with an ionization voltage of 70 eV, the temperatures of the ion source and transmission line were 230 °C and 290 °C, respectively, and a full scanning mode with a mass scanning range of 33–325 amu. The aroma constituents were qualified by comparative analysis of the mass spectrometry data according to the NIST 17 standard library, and semi-quantification was performed by comparing the area of the total ion chromatogram with that of the internal standard.

### Analysis of microbial community

The DNA of the microorganisms present in the samples was extracted using either the CTAB or SDS methods. Following this, the purity of the extracted DNA was detected using agarose gel electrophoresis. The samples were then diluted to a concentration of 1 ng/μL using sterile water. The 16SV34 and ITS1 regions were selected for sequencing using the diluted DNA as a template. Primers 341F and 806R, and the primers ITS1-F and ITS2 were employed for the purpose of PCR amplification. The PCR products were subjected to electrophoresis on an agarose gel at a concentration of 2%. The library was constructed using a library construction kit. Following quantification using the Qubit and qPCR methods, the constructed libraries were quantified and then sequenced using the HiSeq2500 PE250 platform.

The effective tags were obtained by splicing the reads of each sample using FLASH, followed by strict filtering and tags quality control according to the specifications outlined in the Quantitative Insights Into Microbial Ecology (QIIME) protocol. The complete set of effective tags from all samples was clustered using Uparse software, with sequences clustered into operational taxonomic units (OTUs) by default at a 97% similarity. The species annotation analysis was conducted using the Mothur method and the SSU rRNA database. The complexity of the samples was analyzed using the α-diversity indices, which were calculated with the QIIME software.

### Statistical analysis

All analyses were conducted in triplicate, and the resulting data were presented as mean ± standard deviation. One-way analysis of variance (ANOVA) and Duncan’s multiple comparison test were employed by IBM SPSS Statistics 25 (IBM Corp., Armonk, NY, USA) to assess the discrepancies between samples^69^. The odor activity value (OAV) was the ratio of the volatiles concentration to the volatiles odor threshold. Non-metric multidimensional scaling (NMDS) was conducted using the OmicShare tool (https://www.omicshare.com/tools). Pearson correlation analysis was conducted using the R to establish a correlation between the aroma constituents and microorganisms.

## Electronic supplementary material

Below is the link to the electronic supplementary material.

Supplementary Material 1

## Data Availability

The original contributions presented in this study are included in the article/supplementary material. Further inquiries can be directed to the corresponding author(s). The datasets generated and analyzed during the current study are available in the NCBI Sequence Read Archive repository under the project accession number PRJNA1256244 [http://www.ncbi.nlm.nih.gov/bioproject/1256244].

## References

[1] Delnevo, C. D., Giovenco, D. P., Ambrose, B. K., Corey, C. G. & Conway, K. P. Preference for flavoured cigar brands among youth, young adults and adults in the USA. *Tob. Control* **24**(4), 389–394 (2015). 24721967 10.1136/tobaccocontrol-2013-051408 PMC6469858

[2] Morris, D. S. & Fiala, S. C. Flavoured, non-cigarette tobacco for sale in the USA: an inventory analysis of Internet retailers. *Tob. Control* **24**(1), 101–102 (2015). 23929812 10.1136/tobaccocontrol-2013-051059

[3] Shi, Y. et al. Identification and discrimination of characteristic aroma constituents of different cigar leaves based on static headspace/gas chromatography-ion mobility spectrometry combined with relative odor activity value and multivariate statistical analysis. *J. Instrum. Anal.* **42**(6), 674–683 (2023).

[4] Hu, W. et al. Sensory attributes, chemical and microbiological properties of cigars aged with different media. *Front. Bioeng. Biotechnol.* **11**, 1294667 (2023). 37941725 10.3389/fbioe.2023.1294667 PMC10628719

[5] Lisuma, J. B., Zuberi, Z., Ndakidemi, P. A. & Mbega, E. R. Linking rhizosphere bacterial diversity and soil fertility in tobacco plants under different soil types and cropping pattern in Tanzania: A pilot study. *Heliyon* **6**(7), e04278 (2020). 32671244 10.1016/j.heliyon.2020.e04278 PMC7347649

[6] Wu, Q. et al. Interaction analysis of tobacco leaf microbial community structure and volatiles flavor compounds during cigar stacking fermentation. *Front. Microbiol.* **14**, 1168122 (2023). 37637131 10.3389/fmicb.2023.1168122 PMC10457113

[7] Gluck-Thaler, E. & Slot, J. C. Dimensions of horizontal gene transfer in eukaryotic microbial pathogens. *PLoS Pathog.* **11**(10), e1005156 (2015). 26513155 10.1371/journal.ppat.1005156 PMC4626037

[8] Tian, Y. et al. The fungal leaf endophyte *Paraconiothyrium variabile* specifically metabolizes the host-plant metabolome for its own benefit. *Phytochemistry* **108**, 95–101 (2014). 25446235 10.1016/j.phytochem.2014.09.021

[9] Trinh, C. S. et al. *Paenibacillus pabuli* strain P7S promotes plant growth and induces anthocyanin accumulation in *Arabidopsis thaliana*. *Plant Physiol. Biochem.* **129**, 264–272 (2018). 29906776 10.1016/j.plaphy.2018.06.001

[10] Zhou, J. et al. Endophytic *Pseudomonas* induces metabolic flux changes that enhance medicinal sesquiterpenoid accumulation in *Atractylodes lancea*. *Plant Physiol. Biochem.* **130**, 473–481 (2018). 30081324 10.1016/j.plaphy.2018.07.016

[11] Yang, M. et al. Characterizing the microbial community of Pixian Doubanjiang and analysing the metabolic pathway of major flavour metabolites. *LWT-Food Sci. Technol.* **143**, 111170 (2021).

[12] Zheng, T. et al. Analysis of microbial community, volatile flavor compounds, and flavor of cigar tobacco leaves from different regions. *Front. Microbiol.* **13**, 907270 (2022). 35756070 10.3389/fmicb.2022.907270 PMC9231593

[13] Zong, P. et al. Effects of adding cocoa fermentation medium on cigar leaves in agricultural fermentation stage. *Front. Bioeng. Biotechnol.* **11**, 1251413 (2023). 37662435 10.3389/fbioe.2023.1251413 PMC10469782

[14] Zhang, L., Li, W., Peng, Z. & Zhang, J. Effect of microbial community on the formation of flavor components in cigar tobacco leaves during air-curing. *BMC Microbiol.* **25**(1), 56 (2025). 39891085 10.1186/s12866-025-03774-2 PMC11783773

[15] Zhang, Q. et al. Microbial and enzymatic changes in cigar tobacco leaves during air-curing and fermentation. *Appl. Microbiol. Biot.* **107**, 5789–5801 (2023). 10.1007/s00253-023-12663-5 PMC10439857 37458766

[16] Gao, Y. et al. Diversity of microbial communities in cigar filler leaves with different initial water contents analyzed based on high-throughput sequencing technology. *Front. Microbiol.* **16**, 1508866 (2025). 39990154 10.3389/fmicb.2025.1508866 PMC11845121

[17] Li, W. et al. The dynamics of microbial community structure and metabolic function in different parts of cigar tobacco leaves during air-curing. *Front. Microbiol.* **15**, 1438566 (2024). 39726961 10.3389/fmicb.2024.1438566 PMC11669699

[18] Liu, F. et al. Microbial community and metabolic function analysis of cigar tobacco leaves during fermentation. *Microbiologyopen* **10**(2), e1171 (2021). 33970539 10.1002/mbo3.1171 PMC8483401

[19] Zhang, M. et al. Comparative profiling of microbial communities and volatile organic compounds in fermented wrapper, binder, and filler cigar tobaccos. *Chem. Biol. Technol. Agric.* **11**, 68 (2024).

[20] Pei, Q. et al. Study on quality enhancement during cigar tobacco fermentation *by Staphylococcus nepalensis*: insights into microbial community, volatile substances and sensory evaluation. *Front. Microbiol.* **16**, 1526178 (2025). 40008043 10.3389/fmicb.2025.1526178 PMC11850395

[21] Su, Y. et al. Contribution of pectin-degrading bacteria to the quality of cigar fermentation: An analysis based on microbial communities and physicochemical components. *Front. Microbiol.* **15**, 1481158 (2024). 39611089 10.3389/fmicb.2024.1481158 PMC11604125

[22] Zhang, Q. et al. Effects of a novel microbial fermentation medium produced by *Tremella aurantialba* SCT-F3 on cigar filler leaf. *Front. Microbiol.* **14**, 1267916 (2023). 37808308 10.3389/fmicb.2023.1267916 PMC10556473

[23] Xian, K., Shen, C., Qi, W., Xia, Q. & Chen, Y. Study on the neutral flavour constituents of Yunnan flue-cured tobacco. *Acta Tabacaria Sinica* **1**(2), 1–9 (1992).

[24] Liu, Z. *Research of full flavor flue-cured tobacco variety screening and differences of aroma substance in varieties* (Henan Agricultural University, 2013).

[25] Liu, L. et al. Study on discriminating flue-cured tobacco by volatile compounds related to geographical origin and cultivar. *Asian J. Chem.* **25**(13), 7587–7592 (2013).

[26] Xu, Y. et al. Flavor mystery of Chinese traditional fermented baijiu: The great contribution of ester compounds. *Food Chem.* **369**, 130920 (2022). 34461518 10.1016/j.foodchem.2021.130920

[27] Piornos, J. A. et al. Elucidating the odor-active aroma compounds in alcohol-free beer and their contribution to the worty flavor. *J. Agric. Food Chem.* **68**(37), 10088–10096 (2020). 32799537 10.1021/acs.jafc.0c03902 PMC7499417

[28] Li, C. et al. A comparative study on aroma component characteristics of tobacco leaves from Sichuan and Shandong based on 51 flue-cured tobacco germplasms. *Chin. Tobacco Sci.* **42**(5), 7–14 (2021).

[29] Wu, X. et al. Study on the correlation between the dominant microflora and the main flavor substances in the fermentation process of cigar tobacco leaves. *Front. Microbiol.* **14**, 1267447 (2023). 38075898 10.3389/fmicb.2023.1267447 PMC10699171

[30] Zhang, M. et al. Integrated characterization of filler tobacco leaves: HS–SPME–GC–MS, E-nose, and microbiome analysis across different origins. *Bioresour. Bioprocess.* **11**(1), 11 (2024). 38647645 10.1186/s40643-024-00728-w PMC10992047

[31] Tso, T. C. *Physiology and biochemistry of tobacco plants* (Dowden, Hutchinson & Ross, Inc., 1972).

[32] Chen, Y. et al. Influences of feed fermentation on chemical compositions and bacterial diversity of cigar filler tobacco. *Acta Tabacaria Sinica* **29**(1), 106–115 (2023).

[33] Johnson, R. R. & Nicholson, J. A. The Structure, chemistry, and synthesis of solanone. A new anomalous terpenoid ketone from tobacco. *J. Org. Chem.* **30**(9), 2918–2921 (1965).

[34] Hou, Y. et al. Regional characteristic analysis of chemical components and sensory quality of Hongda tobacco in Yunnan province. *J. Yunnan Agric. Univ. (Nat. Sci.)* **32**(4), 659–667 (2017).

[35] Zhou, F., Qi, W., Zhang, H., Liu, D. & Zhao, P. Isophorone oxide and its role in cigarette favoring. *Tobacco Sci. Technol.* **30**(1), 29 (1997).

[36] Wang, H. et al. Correlation study on microbial communities and volatile flavor compounds in cigar tobacco leaves of diverse origins. *Appl. Microbiol. Biotechnol.* **108**, 236 (2024). 38407656 10.1007/s00253-024-13032-6 PMC10896874

[37] Di Giacomo, M. et al. Microbial community structure and dynamics of dark fire-cured tobacco fermentation. *Appl. Environ. Microbiol.* **73**(3), 825–837 (2007). 17142368 10.1128/AEM.02378-06 PMC1800767

[38] Zhao, M., Liu, Y., Li, F., Wang, B. & Liu, G. Identification of dominant and fragrance-enhancing microorganisms of tobacco leaves during ripening. *Acta Microbiol. Sin.* **49**(5), 624–630 (2009). 19637570

[39] Yao, L. et al. Application of yeast in plant-derived aroma formation from cigar filler leaves. *Front. Bioeng. Biotech.* **10**, 1093755 (2022). 10.3389/fbioe.2022.1093755 PMC9815610 36619396

[40] Su, Y. et al. Biodegradation of lignin and nicotine with white rot fungi for the delignification and detoxification of tobacco stalk. *BMC Biotechnol.* **16**(1), 81 (2016). 27871279 10.1186/s12896-016-0311-8 PMC5117543

[41] Macris, B. J. Production and characterization of cellulase and β-glucosidase from a mutant of Alternaria. *Appl. Environ. Microbiol.* **47**(3), 560–565 (1984). 16346494 10.1128/aem.47.3.560-565.1984 PMC239720

[42] Zhang, G. et al. Characterization and succession analysis of bacterial community diversity in different fermentation cycles of Hainan H382 cigar leaf. *Acta Tabacaria Sinica* **27**(2), 117–126 (2021).

[43] Costa, O. Y. A., de Hollander, M., Pijl, A., Liu, B. & Kuramar, E. E. Cultivation-independent and cultivation-dependent metagenomes reveal genetic and enzymatic potential of microbial community involved in the degradation of a complex microbial polymer. *Microbiome* **8**, 76 (2020). 32482164 10.1186/s40168-020-00836-7 PMC7265232

[44] Lakshmi, H. P. et al. Molecular characterization of α-amylase from *Staphylococcus aureus*. *Bioinformation* **9**(6), 281–285 (2013). 23559746 10.6026/97320630009281 PMC3607186

[45] Wang, X. et al. Multi-omics reveals the phyllosphere microbial community and material transformations in cigars. *Front. Microbiol.* **15**, 1436382 (2024). 39144227 10.3389/fmicb.2024.1436382 PMC11322134

[46] Banožić, M., Jokić, S., Ačkar, Đ, Blažić, M. & Šubarić, D. Carbohydrates-key players in tobacco aroma formation and quality determination. *Molecules* **25**(7), 1734 (2020). 32283792 10.3390/molecules25071734 PMC7181196

[47] Geng, Z. et al. Aroma precursors of cigars from different tobacco parts and origins, and their correlations with sensory characteristics. *Front. Plant. Sci.* **14**, 1264739 (2023). 38192690 10.3389/fpls.2023.1264739 PMC10773810

[48] Tao, Y., García, J. F. & Sun, D. W. Advances in wine aging technologies for enhancing wine quality and accelerating wine aging process. *Crit. Rev. Food Sci. Nutr.* **54**(6), 817–835 (2014). 24345051 10.1080/10408398.2011.609949

[49] Dzialo, M. C., Park, R., Steensels, J., Lievens, B. & Verstrepen, K. J. Physiology, ecology and industrial applications of aroma formation in yeast. *FEMS Microbiol. Rev.* **41**(Supp_1), S95–S128 (2017). 28830094 10.1093/femsre/fux031 PMC5916228

[50] Zhong, W. et al. Degradation of nicotine in tobacco waste extract by newly isolated *Pseudomonas* sp.. *ZUTSKD. Bioresour. Technol.* **101**(18), 6935–6941 (2010). 20434329 10.1016/j.biortech.2010.03.142

[51] Mu, Y. et al. Bacterial catabolism of nicotine: catabolic strains, pathways and modules. *Environ. Res.* **183**, 109258 (2020). 32311908 10.1016/j.envres.2020.109258

[52] Liu, T. et al. Phyllosphere microbial community of cigar tobacco and its corresponding metabolites. *Front. Microbiol.* **13**, 1025881 (2022). 36439836 10.3389/fmicb.2022.1025881 PMC9691965

[53] Kandel, S. L., Joubert, P. M. & Doty, S. L. Bacterial endophyte colonization and distribution within plants. *Microorganisms* **5**(4), 77 (2017). 29186821 10.3390/microorganisms5040077 PMC5748586

[54] Zhou, Y. et al. Soil fertility and crop production are fostered by micro-nano bubble irrigation with associated changes in soil bacterial community. *Soil. Biol. Biochem.* **141**, 107663 (2020).

[55] Wei, Y. et al. Phyllosphere fungal communities of rubber trees exhibited biogeographical patterns, but not bacteria. *Environ. Microbiol.* **24**(8), 3777–3790 (2022). 35001480 10.1111/1462-2920.15894

[56] Zhang, G., Shen, Z. & Fu, G. Geo-distribution patterns of soil fungal community of *Pennisetum flaccidum* in Tibet. *J. Fungi* **8**(11), 1230 (2022). 10.3390/jof8111230 PMC9699603 36422051

[57] Xing, L. et al. Influence of association network properties and ecological assembly of the foliar fugal community on crop quality. *Front. Microbiol.* **13**, 783923 (2022). 35479639 10.3389/fmicb.2022.783923 PMC9037085

[58] Marissa, R. L. & Christine, V. H. Plant and soil drivers of whole-plant microbiomes: Variation in switchgrass fungi from coastal to mountain sites. *Phytobiomes J.* **5**(1), 69–79 (2021).

[59] Beattie, G. A. Water relations in the interaction of foliar bacterial pathogens with plants. *Annu. Rev. Phytipathol.* **49**, 533–555 (2011). 10.1146/annurev-phyto-073009-114436 21438680

[60] Bao, L. et al. Seasonal variation of epiphytic bacteria in the phyllosphere of *Gingko biloba*, *Pinus bungeana* and *Sabina chinensis*. *FEMS Microbiol. Ecol.* **96**(3), fiaa017 (2020). 32005997 10.1093/femsec/fiaa017

[61] Andrews, J. H. & Robin, F. H. The ecology and biogeography of microorganisms on plant surfaces. *Annu. Rev. Phytipathol.* **38**, 145–180 (2000). 10.1146/annurev.phyto.38.1.145 11701840

[62] Jackson, C. R. & Denney, W. C. Annual and seasonal variation in the phyllosphere bacterial community associated with leaves of the southern magnolia (*Magnolia grandiflora*). *Microb. Ecol.* **61**(1), 113–122 (2011). 20809288 10.1007/s00248-010-9742-2

[63] Finkel, O. M. et al. Distance-decay relationships partially determine diversity patterns of phyllosphere bacteria on Tamrix trees across the Sonoran Desert. *Appl. Environ. Microb.* **78**(17), 6187–6193 (2012). 10.1128/AEM.00888-12 PMC3416633 22752165

[64] Jia, Y. et al. Development of *Candida autochthonous* starter for cigar fermentation via dissecting the microbiome. *Front. Microbiol.* **14**, 1138877 (2023). 36910204 10.3389/fmicb.2023.1138877 PMC9998997

[65] Song, W. et al. Effects of *Bacillus altitudinis* inoculants on cigar tobacco leaf fermentation. *Front. Bioeng. BiotechI.* **12**, 1417601 (2024). 10.3389/fbioe.2024.1417601 PMC11264575 39045536

[66] Zheng, T. et al. Effects of inoculation with *Acinetobacter* on fermentation of cigar tobacco leaves. *Front. Microbiol.* **13**, 911791 (2022). 35783443 10.3389/fmicb.2022.911791 PMC9248808

[67] Zhang, Q. et al. Analysis of the structure and metabolic function of microbial community in cigar tobacco leaves in agricultural processing stage. *Front. Microbiol.* **14**, 1230547 (2023). 37637128 10.3389/fmicb.2023.1230547 PMC10448963

[68] Domínguez-Tornay, A., Díaz, A. B., Lasanta, C., Durán-Guerrero, E. & Castro, R. Co-fermentation of lactic acid bacteria and *Saccharomyces cerevisiae* for the production of a probiotic beer: Survival and sensory and analytical characterization. *Food Biosci.* **57**, 103482 (2024).

[69] Zhao, L. & Zhao, D. Hydrolyzed polyacrylamide biotransformation during the formation of anode biofilm in microbial fuel cell biosystem: Bioelectricity, metabolites and functional microorganisms. *Bioresour. Technol.* **360**, 127581 (2022). 35798169 10.1016/j.biortech.2022.127581

