# Publisher Correction: Characterization of aroma profiles and microbial communities of cigar tobacco leaves from different varieties and origins and their correlations analysis

**DOI:** 10.1038/s41598-025-14904-1

**Published:** 2025-08-07

**Authors:** Zhaoliang Geng, Huajun Gao, Zhuokuan Tang, Wenhui Zhu, Tongjing Yan, Beisen Lin, Qi Li, Xianwei Hao, Can Lyu, Bin Cai, Zelin He, Jian Liu

**Affiliations:** 1 Hainan Provincial Branch of China National Tobacco Corporation, Haikou, 571103 China; 2 Hainan Hongta Cigarette Company Limited, Haikou, 571137 China; 3 China Tobacco Zhejiang Industrial Company Limited, Hangzhou, 311500 China; 4 Institute of Tobacco Research, Chinese Academy of Agricultural Sciences, Qingdao, 266101 China; 5 Beijing Life Science Academy, Beijing, 100085 China

Correction to: *Scientific Reports* 10.1038/s41598-025-07310-0, published online 15 July 2025

The Competing interests section in the original version of this Article was incorrect.

"The authors declare no competing interests."

now reads:

"Authors Zhaoliang Geng, Huajun Gao, Tongjing Yan, Beisen Lin, and Bin Cai are employed by the Hainan Provincial Branch of China National Tobacco Corporation. Authors Zhuokuan Tang and Wenhui Zhu are employed by Hainan Hongta Cigarette Company Limited. Authors Qi Li and Xianwei Hao are employed by China Tobacco Zhejiang Industrial Company Limited. Authors Zelin He and Jian Liu are employed by the Institute of Tobacco Research, Chinese Academy of Agricultural Sciences. There are no personal financial interests (stocks or shares, consultation fees or other forms of remuneration, patents or patent applications) that may gain or lose financially through publication. This study received funding from China National Tobacco Corporation, Hainan Provincial Branch of China National Tobacco Corporation, Beijing Life Science Academy Company Limited, Hunan Tobacco Company Chenzhou Company, and China Tobacco Shandong Industrial Company Limited. The funders are not involved in conceptualization, design, data collection, analysis, decision to publish, or preparation of the manuscript. All authors declare no other competing interests."

The original Article has been corrected.

