# Supplementary material for: Characterization of aroma profiles and microbial communities of cigar tobacco leaves from different varieties and origins and their correlations analysis
Source: Sci Rep. 2025 Jul 15;15:25556. doi: 10.1038/s41598-025-07310-0 (PMC12263877; doi:10.1038/s41598-025-07310-0)
Supplement: Supplementary file 1 — Supplementary Material 1 [file 41598_2025_7310_MOESM1_ESM.docx]

Table S1 The aroma constituents of cigar tobacco leaves from different wrapper varieties (μg/kg)

| RT | Compounds | CAS | Odor description | OTH  (mg/m^3^)  ^[1-23]^ | Variety | | |
| --- | --- | --- | --- | --- | --- | --- | --- |
|  |  |  |  |  | AQ1 | AQ2 | AQ3 |
|  | Esters (6) |  |  |  |  |  |  |
| 8.56 | Methyl butanoate | 623-42-7 | Ether, fruit, sweet | 0.03 | 1.45±0.99^a^ | 1.76±0.58^a^ | 1.46±0.04^a^ |
| 9.91 | Methyl 2-methylbutyrate | 868-57-5 | Apple | 0.00015 | 0.30±0.05^a^ | Nd | Nd |
| 13.56 | Isobutyl isobutyrate | 97-85-8 | Fruit | 0.44 | Nd | Nd | 1.02±0.52^a^ |
| 16.98 | Lavender lactone | 1073-11-6 |  | Ukn | Nd | 14.01±1.50^a^ | Nd |
| 28.60 | Dihydroactinidiolide | 17092-92-1 | Coumarin, musk | 0.28 | 161.43±3.99^a^ | 174.23±13.20^ab^ | 192.09±17.04^b^ |
| 36.21 | Norambreinolid | 564-20-5 | Floral | Ukn | 6.94±1.47^a^ | Nd | 9.60±1.06^b^ |
|  | Total |  |  |  | 170.12±3.29^a^ | 190.01±12.03^ab^ | 204.16±18.21^b^ |
|  | Alcohols (6) |  |  |  |  |  |  |
| 30.60 | Viridiflorol | 552-02-3 |  | Ukn | Nd | Nd | 26.48±1.54^a^ |
| 31.70 | Hexa-hydro-farnesol | 6750-34-1 |  | Ukn | 107.01±9.11^a^ | Nd | Nd |
| 33.94 | 3,7,11,15-Tetramethyl-2-hexadecen-1-ol | 102608-53-7 |  | Ukn | 24.40±2.63^ab^ | 26.64±0.59^b^ | 21.48±1.92^a^ |
| 35.13 | 2-Hexyldecanol | 2425-77-6 |  | Ukn | Nd | 4.03±0.24^a^ | Nd |
| 35.80 | Thunbergol | 25269-17-4 |  | Ukn | 17.27±4.88^a^ | 29.73±9.11^b^ | 32.62±2.41^b^ |
| 36.10 | Phytol | 150-86-7 | Flower | 0.64 | 11.23±3.14^a^ | 14.35±3.65^a^ | 11.64±2.26^a^ |
|  | Total |  |  |  | 159.91±18.57^a^ | 74.76±12.40^b^ | 92.22±6.10^b^ |
|  | Acids (1) |  |  |  |  |  |  |
| 17.64 | 3-Methylpentanoic acid | 105-43-1 | Pear, fruit | Ukn | Nd | 9.51±1.79^a^ | 58.85±8.53^b^ |
|  | Total |  |  |  | Nd | 9.51±1.79^a^ | 58.85±8.53^b^ |
|  | Aldehydes (7) |  |  |  |  |  |  |
| 10.14 | Prenal | 107-86-8 |  | Ukn | 1.21±0.05^a^ | 0.83±0.09^b^ | 0.32±0.17^c^ |
| 10.56 | Hexanal | 66-25-1 | Apple, fruit | 0.23 | 5.49±0.57^ab^ | 6.45±0.28^b^ | 4.65±0.71^a^ |
| 11.96 | trans-2-Hexenal | 6728-26-3 | Fresh, sweet | 0.0031 | Nd | 2.75±0.24^a^ | Nd |
| 15.07 | Benzaldehyde | 100-52-7 | Sugar, cherry | 0.0850 | 8.98±0.37^b^ | 6.11±0.11^a^ | 5.82±0.26^a^ |
| 15.99 | Octanal | 124-13-0 | Fat, soap, lemon | 0.17 | 1.90±0.32^a^ | Nd | Nd |
| 18.65 | Nonanal | 124-19-6 | Citrus, citrus | 0.0031 | 45.19±17.84^a^ | 59.96±9.51^a^ | 35.69±4.76^a^ |
| 21.11 | Decanal | 112-31-2 | Orange peel | 0.0026 | 7.69±1.03^a^ | 16.14±0.37^b^ | 11.00±1.56^c^ |
|  | Total |  |  |  | 70.46±18.14^ab^ | 92.24±9.73^b^ | 57.48±6.26^a^ |
|  | Ketones (19) |  |  |  |  |  |  |
| 5.25 | Acetone | 67-64-1 | Fruit, hay | 2.00 | 13.22±1.56^a^ | Nd | Nd |
| 6.14 | Butenone | 78-94-4 | Sweet | 0.50 | Nd | 1.37±0.25^a^ | 0.61±0.19^b^ |
| 6.24 | Butanone | 78-93-3 | Ether | 1.30 | 1.52±0.36^a^ | Nd | Nd |
| 8.00 | Propione | 96-22-0 | Ether | 3.00 | 1.08±0.29^a^ | Nd | Nd |
| 15.47 | Methyl heptenone | 110-93-0 | Citrus, strawberry | 0.0189 | 42.42±3.51^ab^ | 45.47±3.08^b^ | 38.34±2.83^a^ |
| 19.25 | Isophorone | 78-59-1 | Cedarwood, spice | 0.0017 | 1.43±0.15^a^ | Nd | 2.12±0.14^b^ |
| 19.54 | 5-Ethyl-6-methyl-3(E)-hepten-2-one | 57283-79-1 |  | Ukn | Nd | 3.38±1.81^a^ | Nd |
| 19.74 | Ketoisophorone | 1125-21-9 | Citrus, honey | Ukn | 8.33±0.99^a^ | 10.84±0.72^b^ | 9.86±0.71^ab^ |
| 20.35 | Dihydrooxophorone | 20547-99-3 |  | Ukn | Nd | 1.60±0.19^a^ | 1.81±0.08^a^ |
| 21.73 | 4,8-Dimethylnona-3,7-dien-2-one | 817-88-9 |  | Ukn | Nd | 8.90±2.84^a^ | Nd |
| 25.29 | Solanone | 54868-48-3 |  | Ukn | 39.59±6.77^a^ | Nd | Nd |
| 25.53 | Hexahydropseudoionone | 1604-34-8 |  | Ukn | Nd | 111.28±3.71^a^ | Nd |
| 26.35 | Dihydro-β-ionone | 17283-81-7 | Floral | 0.0017 | Nd | 34.90±1.02^a^ | 31.39±1.02^b^ |
| 26.56 | Geranyl acetone | 689-67-8 | Fresh, rose | Ukn | 803.02±9.94^a^ | 978.89±61.82^b^ | 698.11±56.13^c^ |
| 28.89 | Megastigmatrienone | 38818-55-2 | Sweet, tobacco | 10.04 | 68.99±9.36^a^ | 181.27±5.58^b^ | 29.74±3.15^c^ |
| 29.16 | Pseudoionone | 141-10-6 |  | Ukn | 23.61±1.12^a^ | 34.15±2.03^b^ | 30.85±3.19^b^ |
| 31.96 | Nootkatone | 4674-50-4 |  | Ukn | 112.01±11.71^a^ | Nd | Nd |
| 33.25 | Solavetivone | 54878-25-0 |  | Ukn | 11.35±1.69^a^ | Nd | Nd |
| 34.32 | Farnesyl acetone | 1117-52-8 | Ether, flower | Ukn | 127.20±20.41^a^ | 140.95±8.39^a^ | 132.48±11.45^a^ |
|  | Total |  |  |  | 1253.76±33.77^a^ | 1553.00±64.60^b^ | 976.30±71.10^c^ |
|  | Nitrogen heterocycles (13) |  |  |  |  |  |  |
| 9.00 | 1-Methylpyrrole | 96-54-8 |  | Ukn | 1.70±0.18^a^ | 1.86±0.49^a^ | Nd |
| 9.19 | Pyridine | 110-86-1 | Fatty | 0.32 | 2.00±0.19^a^ | Nd | 1.02±0.32^b^ |
| 11.24 | Methylpyrazine | 109-08-0 | Cocoa, green | 1.90 | 0.87±0.04^a^ | Nd | Nd |
| 11.50 | 2-Methylpyrrole | 636-41-9 |  | Ukn | 9.07±1.21^a^ | 6.67±0.45^b^ | Nd |
| 13.60 | 2,5-Dimethylpyrazine | 123-32-0 | Cocoa, roasted nut | 1.82 | 0.80±0.16^a^ | Nd | Nd |
| 13.62 | 2,6-Dimethylpyrazine | 108-50-9 | Nut, cocoa | 1.72 | Nd | Nd | 0.38±0.01^a^ |
| 16.17 | Nicotinonitrile | 100-54-9 |  | Ukn | 41.38±3.79^b^ | 36.49±1.78^ab^ | 33.97±1.32^a^ |
| 16.91 | Methylpyrrolidone | 872-50-4 |  | Ukn | 19.72±1.94^a^ | Nd | Nd |
| 18.91 | 3-Acetopyridine | 350-03-8 |  | Ukn | 588.41±50.16^a^ | 382.56±13.09^b^ | 410.85±39.74^b^ |
| 23.58 | Ethylmethylmaleimide | 20189-42-8 |  | Ukn | 24.53±1.42^a^ | Nd | 13.81±0.48^b^ |
| 26.41 | Myosmine | 532-12-7 |  | Ukn | 335.63±26.06^a^ | 90.61±5.14^b^ | 139.69±11.56^c^ |
| 28.57 | Isonicoteine | 581-50-0 |  | Ukn | 307.88±56.90^a^ | Nd | 222.12±29.48^a^ |
| 31.57 | Cotinine | 486-56-6 |  | Ukn | 16.07±1.09^a^ | Nd | Nd |
|  | Total |  |  |  | 1348.05±121.72^a^ | 518.18±11.54^b^ | 821.84±80.77^c^ |
|  | Alkenes (8) |  |  |  |  |  |  |
| 12.82 | 1-Acetylcyclohexene | 932-66-1 |  | Ukn | 7.70±0.31^a^ | 7.24±0.20^a^ | 6.27±1.06^a^ |
| 18.52 | β-Ocimene | 13877-91-3 |  | Ukn | Nd | 1.90±0.29^a^ | Nd |
| 27.75 | Valencen | 4630-07-3 |  | Ukn | 24.49±2.31^a^ | 24.24±0.99^a^ | 21.82±2.06^a^ |
| 29.73 | Bicylogermacrene | 24703-35-3 |  | Ukn | Nd | Nd | 91.73±6.40^a^ |
| 31.70 | 1-Nonadecene | 18435-45-5 |  | Ukn | Nd | 135.89±4.03^a^ | Nd |
| 31.81 | 1-Tetradecene | 1120-36-1 |  | Ukn | Nd | 20.01±1.89^a^ | 17.54±1.70^a^ |
| 33.73 | Neophytadiene | 504-96-1 | Fresh | Ukn | 20.88±2.40^a^ | Nd | 18.56±0.45^a^ |
| 34.71 | Cembrene | 1898-13-1 |  | Ukn | 20.62±3.80^a^ | 37.86±3.67^b^ | 35.64±6.53^b^ |
|  | Total |  |  |  | 73.69±5.20^a^ | 227.14±9.54^b^ | 191.56±16.80^c^ |
|  | Total volatiles |  |  |  | 3075.99±171.83^a^ | 2664.83±47.90^b^ | 2396.23±161.72^b^ |

Data are the means ± standard deviation (n=3)

Values in the same row with different letters indicate significant differences (*P* < 0.05) by Duncan’s tests

OTH, odor threshold

Ukn, unknown

ND, not detected

Odor description reported in the http://www.flavornet.org/flavornet.html and http://www.thegoodscentscompany.com/search2.html

References:

1. Nagata, Y. Odor intensity and odor threshold value. *Noise and Vibration Environmental Management Bureau. Ministry of the Environment, Government of Japan*,

41(2): 118-127(2003).

1. Blank, I., Sen, A. & Grosch, W. Aroma impact compounds of Arabica and Robusta coffee. Qualitative and quantitative investigations. 1991.doi: http://dx.doi.org/.
2. van Gemert, L.J. Compilations of odour threshold values in air,water and other media. *Science Press*, 2018.
3. Tamura, H., Nakamoto, H., Yang, R.H., Sugisawa, H. Characteristic Aroma Compounds in Green Algae (Ulva pertusa) Volatiles. *Nippon Shokuhin Kagaku Kogaku*

*Kaishi.* 42(11):887-891(1995). doi:10.3136/nskkk.42.887.

1. Laska, M. Olfactory Perception of 6 Amino Acids by Human Subjects. *Chemical Senses*, 35(4):279-287(2010). doi:10.1093/chemse/bjq017.
2. Yang, R., Sugisawa, H., Nakatani, H., Tamura, H., Takagi, N. Comparison of Odor Quality in Peel Oils of Acid Citrus. *J. Food, SCI. Tech. MYS.* 1992, 39:16-

24(1992). doi:10.3136/NSKKK1962.39.16.

1. Cometto-Muiz, J.E. Odor detection by humans of lineal aliphatic aldehydes and helional as gauged by dose-response functions. *Chemical Senses*, 35(4)(2010): 289-

299. doi:10.1093/chemse/bjq018.

1. Martirosyan, A.S. Maximum Permissible Concentration of Methyl Vinyl Ketone in the Air of a Working Area. Tr. Klin. Otd. Nauch.-Issued. Inst. Gig. Profzabol.

1970(1).

1. Dravnieks, A. A building-block model for the characterization of odorant molecules and their odors. *ANN. NY. ACAD. SCI.* 237(Odors: Evaluation):144-163(2010).

doi:10.1111/j.1749-6632.1974.tb49851.x.

1. Delfini, C. Observations expérimentales sur l'origine et la disparition de l'alcool benzylique et de l'aldéhyde benzoïque dans les moûts et les vins. *Bulletin de l'OIV*.

60(675-676):463-473(1987). http://pascal-francis.inist.fr/vibad/index.php?action=getRecordDetail&idt=8194359.

1. Ziemer, P.D. & Anagnostou, W.T. Study of odor qualification of solvents used in coating compositions. *J. COAT. TECHNOL. RES*. 2000. doi:10.1007/BF02698028.
2. Brenna, E., Fuganti, C., Serra, S & Kraft, P. Optically active ionones and derivatives: preparation and olfactory properties. EUR. J. ORG. CHEM. (6):967-

978(2002).

1. Engen, T., Cain, W.S & Rovee, C.K. Direct scaling of olfaction in the newborn infant and the adult human observer. InTheories of odors and odor measurement

272-294(1968). Istanbul: Robert College.

1. Fors, S.M. & Olofsson, B.K. Alkylpyrazines, volatiles formed in the Maillard reaction. I. Determination of odour detection thresholds and odour intensity functions

by dynamic olfactometry. *Chemical Senses.* (3):3(1985). doi:10.1093/chemse/10.3.287.

1. Wagner, R., Czerny, M., Bielohradsky, J. & Grosch, W. Structure-odour-activity relationships of alkylpyrazines. *Zeitschrift für Lebensmitteluntersuchung* und-

forschung A. 208:308-316(1999). doi:10.1007/s002170050422.

1. Nagy, G.Z. The odor impact model. Journal of the Air & Waste Management Association. 1991, 41(10):1360-1362.
2. Ueno, H., Amano, S., Merecka, B., [Kośmider](javascript:;), J. Difference in the odor concentrations measured by the triangle odor bag method and dynamic olfactometry. *Water*

*Science & Technology A Journal of the International Association on Water Pollution Research.* 59(7):1339(2009). doi:10.2166/wst.2009.112.

1. Pieter H.Punter. Measurement of human olfactory thresholds for several groups of structurally related compounds. *Chemical Senses,* 1983, 7(3-4):215-235(1983).

doi:10.1093/chemse/7.3-4.215.

1. Schieberle, P., Ehrmeier, H. & Grosch, W. Aromastoffe aus dem säurekatalysierten Abbau von Citral. *Zeitschrift für Lebensmittel-Untersuchung und Forschung.*

187:35-39(1988). doi:10.1007/BF01454320.

1. Matsuura, A. & Komatsu, K. Efficient synthesis of benzene and planar cyclooctatetraene fully annelated with bicyclo[2.1.1]hex-2-ene. *Journal of the American*

*Chemical Society*, 123(8):1768(2001). doi:10.1021/ja015156t.

1. Groningen， R. Biophysics of the sense of smell. 1958.
2. Roland， S. & Cain, W.S. Making Scents: Dynamic Olfactometry for Threshold Measurement. *Chemical Senses.* (2):109[2025-04-07]. doi:10.1093/chemse/bjp088.
3. Gemert, L.J.V. Compilations of odour threshold values in air, water and other media. 2003.

Table S2 The aroma constituents of cigar tobacco leaves from different filler varieties (μg/kg)

| RT | Compounds | CAS | Odor description | OTH  (mg/m^3^)  ^[1-23]^ | Variety | | |
| --- | --- | --- | --- | --- | --- | --- | --- |
|  |  |  |  |  | QDQX-1 | QDQX-2 | QDQX-3 |
|  | Esters (8) |  |  |  |  |  |  |
| 8.55 | Methyl butanoate | 623-42-7 | Ether, fruit, sweet | 0.03 | 1.35±0.09^a^ | 0.83±0.10^b^ | 1.55±0.08^c^ |
| 10.20 | Ethyl acetate | 141-78-6 | Pineapple | 4.30 | 0.66±0.17^a^ | Nd | Nd |
| 13.63 | Isobutyl isobutyrate | 97-85-8 | Fruit | 0.44 | Nd | 0.84±0.24^a^ | 0.72±0.19^a^ |
| 17.00 | Lavender lactone | 1073-11-6 |  | Ukn | 54.71±4.00^a^ | 36.84±3.81^b^ | 26.47±1.92^c^ |
| 20.69 | δ-Decenolactone | 54814-64-1 |  | Ukn | Nd | 22.04±1.04^a^ | Nd |
| 28.69 | Dihydroactinidiolide | 17092-92-1 | Woody | 0.28 | Nd | 151.35±12.10^a^ | 208.18±18.14^b^ |
| 36.21 | Norambreinolid | 564-20-5 | Floral | Ukn | 5.91±0.76^a^ | 2.39±0.22^b^ | 2.68±0.58^b^ |
| 38.49 | Farnesyl acetate | 4128-17-0 |  | Ukn | 1.37±0.07^a^ | Nd | 1.40±0.49^a^ |
|  | Total |  |  |  | 63.99±4.03^a^ | 214.28±9.67^b^ | 241.01±17.09^c^ |
|  | Alcohols (13) |  |  |  |  |  |  |
| 17.84 | 3-Acetopropanol | 1071-73-4 |  | Ukn | Nd | Nd | 13.16±1.49^a^ |
| 21.73 | 4,8-Dimethylnonanol | 33933-80-1 |  | Ukn | Nd | 8.97±0.98^a^ | 30.20±2.05^b^ |
| 25.70 | Methylundecylcarbinol | 1653-31-2 |  | Ukn | Nd | Nd | 80.59±2.58^a^ |
| 25.71 | 6,10,14-trimethylpentadecan-2-ol | 69729-17-5 |  | Ukn | 72.46±5.27^a^ | Nd | Nd |
| 28.74 | Nerolidol | 7212-44-4 |  | Ukn | 60.09±7.64^a^ | Nd | 59.77±5.79^a^ |
| 28.83 | Phytol | 150-86-7 | Flower | 0.64 | 12.95±1.70^a^ | 8.53±0.81^b^ | 49.58±1.93^c^ |
| 30.13 | Hexa-hydro-farnesol | 6750-34-1 |  | Ukn | 75.53±5.28^a^ | 77.14±5.27^a^ | 99.61±2.42^b^ |
| 30.85 | Dodecanol | 112-53-8 | Earthy, soapy | 0.41 | Nd | 11.51±1.91^a^ | Nd |
| 33.73 | 3,7,11,15-Tetramethyl-2-hexadecen-1-ol | 102608-53-7 |  | Ukn | Nd | 18.05±1.88^a^ | 18.08±1.77^a^ |
| 34.64 | Isophytol | 505-32-8 |  | Ukn | Nd | 6.30±0.34^a^ | Nd |
| 34.70 | Thunbergol | 25269-17-4 |  | Ukn | 11.34±0.91^a^ | 6.69±0.77^b^ | 20.44±2.02^c^ |
| 35.12 | 2-Hexyldecanol | 2425-77-6 |  | Ukn | 7.17±0.35^a^ | 7.63±0.46^a^ | Nd |
| 35.39 | Geranylgeraniol | 24034-73-9 |  | Ukn | Nd | 2.84±0.26^a^ | 3.65±0.46^b^ |
|  | Total |  |  |  | 239.54±16.77^a^ | 147.68±10.07^b^ | 375.09±8.30^c^ |
|  | Acids (1) |  |  |  |  |  |  |
| 17.58 | 3-Methylpentanoic acid | 105-43-1 | Savory | Ukn | 27.47±3.28^a^ | Nd | 10.18±3.61^b^ |
|  | Total |  |  |  | 27.47±3.28^a^ | Nd | 10.18±3.61^b^ |
|  | Aldehydes (10) |  |  |  |  |  |  |
| 5.93 | Methacrylaldehyde | 78-85-3 | Floral | 0.0250 | 2.88±0.99^a^ | 1.14±0.08^b^ | 1.03±0.23^b^ |
| 7.19 | Crotonaldehyde | 4170-30-3 |  | Ukn | Nd | 1.05±0.11^a^ | Nd |
| 8.02 | Valeraldehyde | 110-62-3 | Almond, green | 0.85 | 3.57±0.45^a^ | 1.55±0.34^b^ | Nd |
| 10.12 | Prenal | 107-86-8 |  | Ukn | 10.16±1.21^a^ | 5.88±0.74^b^ | 3.65±0.10^c^ |
| 10.54 | Hexanal | 66-25-1 | Apple, fresh | 0.0340 | 19.68±1.14^a^ | 8.81±1.32^b^ | 7.15±0.38^b^ |
| 15.05 | Benzaldehyde | 100-52-7 | Sugar, cherry | 0.0850 | 14.61±0.25^b^ | 14.20±1.07^b^ | 9.96±0.27^a^ |
| 16.12 | Octanal | 124-13-0 | Fat, soap, lemon | 0.17 | Nd | 1.93±0.19^a^ | 2.63±0.30^b^ |
| 18.65 | Nonanal | 124-19-6 | Fatty | 0.0031 | Nd | Nd | 68.31±2.84^a^ |
| 21.24 | Decanal | 112-31-2 | Orange peel, soap | 0.0026 | Nd | 18.12±0.97^a^ | 17.55±0.65^a^ |
| 21.92 | Neral | 106-26-3 | Lemon | 0.0088 | 9.06±0.62^a^ | Nd | Nd |
|  | Total |  |  |  | 59.96±3.59^a^ | 52.67±1.48^b^ | 110.29±4.00^c^ |
|  | Ketones (16) |  |  |  |  |  |  |
| 5.24 | Acetone | 67-64-1 | Fruit, hay | 2.00 | 32.19±2.99^a^ | 17.73±0.59^b^ | Nd |
| 6.12 | Butenone | 78-94-4 | Sweet | 0.50 | 9.38±2.38^a^ | 4.05±0.76^b^ | 3.22±0.32^b^ |
| 15.47 | Methyl heptenone | 110-93-0 | Citrus, strawberry | 0.018 | 83.86±7.87^a^ | 52.73±4.57^b^ | 41.51±3.48^b^ |
| 19.25 | Isophorone | 78-59-1 | Cedarwood, spice | 0.0017 | Nd | Nd | 1.34±0.11^a^ |
| 19.55 | 5-Ethyl-6-methyl-3(E)-hepten-2-one | 57283-79-1 |  | Ukn | 4.93±1.33^a^ | 1.92±0.39^b^ | Nd |
| 19.74 | Ketoisophorone | 1125-21-9 | Citrus, honey | 0.0250 | 12.75±0.73^a^ | 9.82±0.87^b^ | 8.55±0.79^b^ |
| 20.35 | Dihydrooxophorone | 20547-99-3 |  | Ukn | 2.20±0.15^a^ | Nd | 1.67±0.15^a^ |
| 21.73 | 4,8-Dimethylnona-3,7-dien-2-one | 817-88-9 |  | Ukn | 12.13±0.54^a^ | Nd | 10.41±2.34^a^ |
| 25.00 | Solanone | 54868-48-3 |  | Ukn | Nd | Nd | 41.68±1.55^a^ |
| 25.55 | Hexahydropseudoionone | 1604-34-8 |  | Ukn | 260.63±10.57^b^ | 226.92±23.16^a^ | 273.30±10.38^b^ |
| 26.68 | Geranyl acetone | 3796-70-1 | Fruit, floral | 0.06 | Nd | 1205.69±143.46^a^ | Nd |
| 28.24 | cis-ψ-Ionone | 13927-47-4 |  | Ukn | Nd | 83.19±10.33^a^ | Nd |
| 28.88 | Megastigmatrienone | 38818-55-2 | Sweet, tobacco | 10.04 | Nd | Nd | 6.65±0.65^a^ |
| 29.22 | Pseudoionone | 141-10-6 |  | Ukn | Nd | 93.25±11.68^a^ | 72.94±4.99^b^ |
| 31.20 | Methyl tridecyl ketone | 2345-28-0 |  | Ukn | 35.66±1.44^a^ | Nd | Nd |
| 34.33 | Farnesyl acetone | 1117-52-8 | Ether, flower | Ukn | 358.53±22.53^a^ | 331.90±30.06^a^ | 260.23±26.61^b^ |
|  | Total |  |  |  | 812.27±25.76^a^ | 2027.21±95.90^b^ | 721.50±35.58^a^ |
|  | Phenols (1) |  |  |  |  |  |  |
| 14.11 | 3,5-Dimethylphenol | 108-68-9 | Balsamic, coffee | 0.00004 | 8.39±0.62^a^ | 7.83±0.56^a^ | Nd |
|  | Total |  |  |  | 8.39±0.62^a^ | 7.83±0.56^a^ | Nd |
|  | Nitrogen heterocycles (10) |  |  |  |  |  |  |
| 9.18 | Pyridine | 110-86-1 | Rancid | 0.32 | Nd | 0.93±0.17^a^ | Nd |
| 11.24 | Methylpyrazine | 109-08-0 | Cocoa, green | 1.90 | Nd | 0.67±0.12^a^ | Nd |
| 11.48 | 2-Methylpyrrole | 636-41-9 |  | Ukn | 57.43±4.37^a^ | Nd | 17.64±1.54^b^ |
| 11.53 | 3-Methylpyrrole | 616-43-3 |  | Ukn | Nd | 34.45±1.73^a^ | Nd |
| 12.34 | 2,5-Dimethylpyrrole | 625-84-3 |  | Ukn | Nd | 127.02±15.06^a^ | 93.13±2.07^b^ |
| 16.16 | Nicotinonitrile | 100-54-9 |  | Ukn | 54.89±1.02^a^ | 43.93±4.10^b^ | 36.94±3.21^c^ |
| 18.94 | 3-Acetopyridine | 350-03-8 |  | Ukn | 699.07±11.51^a^ | 670.89±30.27^a^ | 626.85±65.15^a^ |
| 26.34 | Myosmine | 532-12-7 |  | Ukn | 233.41±19.83^a^ | 224.05±26.94^a^ | 110.43±2.99^b^ |
| 27.39 | Nicotyrine | 487-19-4 |  | Ukn | Nd | Nd | 637.07±125.53^a^ |
| 28.56 | Isonicoteine | 581-50-0 |  | Ukn | 402.42±111.51^a^ | 491.02±96.19^a^ | 209.44±25.68^b^ |
|  | Total |  |  |  | 1447.22±136.86^a^ | 1592.95±74.04^ab^ | 1731.51±163.57^b^ |
|  | Alkenes (4) |  |  |  |  |  |  |
| 12.81 | 1-Acetylcyclohexene | 932-66-1 |  | Ukn | 28.93±2.48^a^ | 26.23±1.26^a^ | 18.73±1.45^b^ |
| 27.93 | Amorphadiene | 92692-39-2 |  | Ukn | 27.54±0.92^a^ | Nd | Nd |
| 31.73 | 1-Nonadecene | 18435-45-5 |  | Ukn | Nd | 193.75±20.35^a^ | Nd |
| 34.71 | Cembrene | 1898-13-1 |  | Ukn | Nd | Nd | 20.92±3.40^a^ |
|  | Total |  |  |  | 56.47±3.06^a^ | 219.98±19.12^b^ | 39.65±2.39^a^ |
|  | Total volatiles |  |  |  | 2715.32±170.89^a^ | 4262.60±47.47^b^ | 3229.33±220.44^c^ |

Data are the means ± standard deviation (n=3)

Values in the same row with different letters indicate significant differences (*P* < 0.05) by Duncan’s tests

OTH, odor threshold

Ukn, unknown

ND, not detected

Odor description reported in the http://www.flavornet.org/flavornet.html and http://www.thegoodscentscompany.com/search2.html

Table S3 The aroma constituents of cigar tobacco leaves from different filler origins (μg/kg)

| RT | Compounds | CAS | Odor description | OTH (mg/m^3^)  ^[1-23]^ | Origins | | | |
| --- | --- | --- | --- | --- | --- | --- | --- | --- |
|  |  |  |  |  | FXQX1 | QDQX1 | QZQX1 | ZCQX1 |
|  | Esters (9) |  |  |  |  |  |  |  |
| 6.54 | Methyl carbonate | 616-38-6 |  | Ukn | Nd | Nd | 0.46±0.11^a^ | Nd |
| 6.74 | Methyl propylate | 554-12-1 | Fruit, rum | 0.35 | 0.35±0.11^a^ | Nd | 0.41±0.11^a^ | Nd |
| 8.56 | Methyl butanoate | 623-42-7 | Ether, fruit, sweet | 0.03 | 1.90±0.80^a^ | 1.35±0.09^a^ | 3.20±0.56^b^ | Nd |
| 9.90 | Methyl 2-methylbutyrate | 868-57-5 | Apple | 0.00015 | Nd | Nd | 0.52±0.07^a^ | Nd |
| 10.20 | Ethyl acetate | 141-78-6 | Pineapple | 4.30 | Nd | 0.66±0.17^a^ | Nd | Nd |
| 16.91 | Lavender lactone | 1073-11-6 |  | Ukn | 7.77±0.30^a^ | 54.71±4.00^b^ | 27.80±1.68^c^ | 22.52±2.35^d^ |
| 28.65 | Dihydroactinidiolide | 17092-92-1 | Woody | 0.28 | 182.36±2.23^a^ | Nd | 120.23±14.52^b^ | 181.47±12.57^a^ |
| 36.23 | Sclareolide | 564-20-5 | Floral | Ukn | 1.84±0.19^a^ | 5.91±0.76^b^ | Nd | Nd |
| 38.49 | Farnesyl acetate | 4128-17-0 |  | Ukn | Nd | 1.37±0.07^a^ | Nd | Nd |
|  | Total |  |  |  | 194.22±2.27^a^ | 63.99±4.03^b^ | 152.63±13.54^c^ | 203.99±10.23^a^ |
|  | Alcohols (10) |  |  |  |  |  |  |  |
| 23.60 | 4,8-Dimethylnonanol | 33933-80-1 |  | Ukn | Nd | Nd | 15.11±1.40^a^ | Nd |
| 25.71 | 6,10,14-Trimethylpentadecan-2-ol | 69729-17-5 |  | Ukn | Nd | 72.46±5.27^a^ | 20.42±0.32^b^ | Nd |
| 28.74 | Nerolidol | 7212-44-4 |  | Ukn | Nd | 60.09±7.64^a^ | Nd | 62.29±7.15^a^ |
| 30.13 | Hexa-hydro-farnesol | 6750-34-1 |  | Ukn | Nd | 75.53±5.28^a^ | Nd | Nd |
| 30.84 | Tetradecanol | 112-72-1 |  | Ukn | 7.82±1.38^a^ | Nd | Nd | Nd |
| 34.00 | 3,7,11,15-Tetramethyl-2-hexadecen-1-ol | 102608-53-7 |  | Ukn | 28.58±3.27^a^ | Nd | Nd | 20.97±2.05^b^ |
| 34.66 | Isophytol | 505-32-8 |  | Ukn | 5.19±0.77^a^ | Nd | Nd | Nd |
| 35.12 | 2-Hexyldecanol | 2425-77-6 |  | Ukn | Nd | 7.17±0.35^a^ | Nd | Nd |
| 35.82 | Thunbergol | 25269-17-4 |  | Ukn | 5.38±0.57^a^ | 11.34±0.91^b^ | Nd | 6.33±3.13^a^ |
| 36.11 | Phytol | 150-86-7 | Flower | 0.64 | 12.20±4.56^a^ | 12.95±1.70^a^ | 9.45±1.80^a^ | Nd |
|  | Total |  |  |  | 59.18±4.01^a^ | 239.54±16.77^b^ | 44.98±3.02^c^ | 89.59±11.42^d^ |
|  | Acids (1) |  |  |  |  |  |  |  |
| 17.58 | 3-Methylpentanoic acid | 105-43-1 | Savory | Ukn | Nd | 27.47±3.28^a^ | Nd | 36.49±2.50^b^ |
|  | Total |  |  |  | Nd | 27.47±3.28^a^ | Nd | 36.49±2.50^b^ |
|  | Aldehydes (11) |  |  |  |  |  |  |  |
| 5.93 | Methacrylaldehyde | 78-85-3 | Floral | 0.0250 | Nd | 2.88±0.99^a^ | Nd | Nd |
| 8.02 | Pentanal | 110-62-3 | Almond, green | 0.85 | Nd | 3.57±0.45^a^ | Nd | Nd |
| 10.12 | Prenal | 107-86-8 |  | Ukn | Nd | 10.16±1.21^a^ | 1.09±0.16^b^ | 1.85±0.29^c^ |
| 10.56 | Hexanal | 66-25-1 | Apple, fresh | 0.034 | 5.12±1.13^a^ | 19.68±1.14^b^ | Nd | 7.39±0.57^c^ |
| 11.99 | trans-2-Hexenal | 6728-26-3 | Fresh, sweet | 0.017 | 1.63±0.16^a^ | Nd | 4.85±0.32^b^ | Nd |
| 15.05 | Benzaldehyde | 100-52-7 | Sugar, cherry | 0.085 | Nd | 14.61±0.25^a^ | 6.56±1.22^b^ | 7.08±0.50^b^ |
| 16.01 | Octanal | 124-13-0 | Fat, soap, lemon | 0.17 | Nd | Nd | 2.37±0.13^a^ | Nd |
| 18.65 | Nonanal | 124-19-6 | Fatty | 0.0031 | 43.00±5.18^a^ | Nd | 61.14±2.29^b^ | 48.16±1.74^a^ |
| 21.11 | Decanal | 112-31-2 | Orange peel, soap | 0.0026 | 11.39±2.52^a^ | Nd | 16.78±1.59^b^ | 14.86±2.72^ab^ |
| 21.92 | Neral | 106-26-3 | Lemon | 0.0088 | Nd | 9.06±0.62^a^ | Nd | Nd |
| 31.53 | Pentadecanal | 2765-11-9 |  | Ukn | Nd | Nd | 12.56±0.54^a^ | Nd |
|  | Total |  |  |  | 61.14±6.53^a^ | 59.96±3.59^a^ | 105.34±2.33^b^ | 79.35±2.73^c^ |
|  | Ketones (18) |  |  |  |  |  |  |  |
| 5.24 | Acetone | 67-64-1 | Fruit, hay | 2.00 | Nd | 32.19±2.99^a^ | Nd | Nd |
| 6.12 | Butenone | 78-94-4 | Sweet | 0.50 | Nd | 9.38±2.38^a^ | Nd | 2.83±0.67^b^ |
| 6.23 | Butanone | 78-93-3 | Ether | 1.30 | Nd | Nd | 2.42±0.58^a^ | Nd |
| 15.48 | Methyl heptenone | 110-93-0 | Citrus, strawberry | 0.0189 | 40.92±3.93^a^ | 83.86±7.87^b^ | 53.68±2.93^c^ | 42.98±2.77^a^ |
| 19.26 | Isophorone | 78-59-1 | Cedarwood, spice | 0.0017 | 1.38±0.21^a^ | Nd | Nd | Nd |
| 19.55 | 5-Ethyl-6-methyl-3(E)-hepten-2-one | 57283-79-1 |  | Ukn | Nd | 4.93±1.33^a^ | Nd | Nd |
| 19.75 | Ketoisophorone | 1125-21-9 | Citrus, honey | 0.0250 | 8.08±0.84^a^ | 12.75±0.73^b^ | 7.70±0.49^a^ | Nd |
| 20.35 | Dihydrooxophorone | 20547-99-3 |  | Ukn | Nd | 2.20±0.15^a^ | 1.73±0.50^ab^ | 1.43±0.22^b^ |
| 21.73 | 4,8-Dimethylnona-3,7-dien-2-one | 817-88-9 |  | Ukn | Nd | 12.13±0.54^a^ | 6.12±1.61^b^ | Nd |
| 25.55 | Hexahydropseudoionone | 1604-34-8 |  | Ukn | Nd | 260.63±10.57^a^ | 85.24±4.44^b^ | 156.96±17.31^c^ |
| 26.58 | Geranyl acetone | 689-67-8 | Fresh rose, floral | Ukn | 856.40±7.87^a^ | Nd | 576.64±22.74^b^ | Nd |
| 27.45 | 5,6-Epoxy-β-ionone | 23267-57-4 |  | Ukn | Nd | Nd | Nd | 119.66±22.95^a^ |
| 28.18 | cis-ψ-Ionone | 13927-47-4 |  | Ukn | 26.63±1.56^a^ | Nd | Nd | Nd |
| 28.91 | Megastigmatrienone | 38818-55-2 | Sweet, tobacco | 10.04 | 124.01±19.27^a^ | Nd | 87.16±2.26^b^ | 68.56±19.04^b^ |
| 29.18 | Pseudoionone | 141-10-6 |  | Ukn | 26.46±0.10^a^ | Nd | 15.35±1.47^b^ | 54.05±4.49^c^ |
| 31.23 | Methyl tridecyl ketone | 2345-28-0 |  | Ukn | 29.46±0.42^a^ | 35.66±1.44^b^ | 30.83±1.74^a^ | Nd |
| 33.26 | Solavetivone | 54878-25-0 |  | Ukn | 7.12±0.05^a^ | Nd | Nd | 9.38±1.59^b^ |
| 34.36 | Farnesyl acetone | 1117-52-8 | Ether, flower | Ukn | 116.56±6.44^a^ | 358.53±22.53^b^ | 94.34±6.89^c^ | 215.92±33.27^d^ |
|  | Total |  |  |  | 1237.03±22.49^a^ | 812.27±25.76^b^ | 961.20±39.92^c^ | 671.77±91.32^d^ |
|  | Phenols (1) |  |  |  |  |  |  |  |
| 14.11 | 3,5-Dimethylphenol | 108-68-9 | Balsamic, coffee | 0.00004 | Nd | 8.39±0.62^a^ | 5.01±0.26^b^ | 6.65±0.24^c^ |
|  | Total |  |  |  | Nd | 8.39±0.62^a^ | 5.01±0.26^b^ | 6.65±0.24^c^ |
|  | Nitrogen heterocycles (9) |  |  |  |  |  |  |  |
| 8.99 | 1-Methylpyrrole | 96-54-8 |  |  | 0.94±0.02^a^ | Nd | 1.87±0.33^b^ | Nd |
| 9.22 | Pyridine | 110-86-1 | Fatty | 0.32 | Nd | Nd | 1.70±0.57^a^ | Nd |
| 11.50 | 2-Methylpyrrole | 636-41-9 |  | Ukn | 6.65±2.10^a^ | 57.43±4.37^b^ | 3.93±0.25^c^ | 1.01±0.18^d^ |
| 12.26 | 2,5-Dimethylpyrrole | 625-84-3 |  | Ukn | Nd | Nd | Nd | 41.46±0.60^a^ |
| 16.17 | Nicotinonitrile | 100-54-9 |  | Ukn | 46.83±12.55^a^ | 54.89±1.02^a^ | 20.58±0.99^a^ | 48.71±1.80^a^ |
| 16.91 | Methylpyrrolidone | 872-50-4 |  | Ukn | 20.25±7.38^a^ | Nd | Nd | Nd |
| 26.38 | Myosmine | 532-12-7 |  | Ukn | 95.05±12.64^a^ | 233.41±19.83^b^ | 82.64±11.46^a^ | 184.83±15.37^c^ |
| 27.45 | B-Nicotyrine | 487-19-4 |  | Ukn | 884.36±95.06^a^ | Nd | 691.05±35.87^b^ | Nd |
| 28.60 | Isonicoteine | 581-50-0 |  | Ukn | 200.04±57.38^a^ | 402.42±111.51^c^ | 70.19±11.09^b^ | 362.38±51.82^c^ |
|  | Total |  |  |  | 1254.11±172.39^c^ | 748.15±125.37^ab^ | 871.97±55.05^b^ | 638.38±67.93^a^ |
|  | Alkenes (5) |  |  |  |  |  |  |  |
| 12.81 | 1-Acetylcyclohexene | 932-66-1 |  | Ukn | Nd | 28.93±2.48^a^ | 6.86±0.57^b^ | 11.35±0.64^c^ |
| 18.52 | β-Ocimene | 13877-91-3 |  | Ukn | Nd | Nd | 1.57±0.30^a^ | Nd |
| 27.93 | Amorpha-4,11-diene | 92692-39-2 |  | Ukn | Nd | 27.54±0.92^a^ | Nd | Nd |
| 29.75 | Bicylogermacrene | 24703-35-3 |  | Ukn | 62.55±1.50^a^ | Nd | Nd | Nd |
| 34.74 | Cembrene | 1898-13-1 |  | Ukn | 6.48±1.61^a^ | Nd | Nd | Nd |
|  | Total |  |  |  | 69.02±0.11^a^ | 56.47±3.06^b^ | 8.43±0.76^c^ | 11.35±0.64^d^ |
|  | Total volatiles |  |  |  | 2874.70±161.61^c^ | 2016.25±159.65^ab^ | 2149.56±109.44^b^ | 1737.56±177.09^a^ |

Data are the means ± standard deviation (n=3)

Values in the same row with different letters indicate significant differences (*P* < 0.05) by Duncan’s tests

OTH, odor threshold

Ukn, unknown

ND, not detected

Odor description reported in the http://www.flavornet.org/flavornet.html and http://www.thegoodscentscompany.com/search2.html
